# 4Cin: A computational pipeline for 3D genome modeling and virtual Hi-C analyses from 4C data

**DOI:** 10.1371/journal.pcbi.1006030

**Published:** 2018-03-09

**Authors:** Ibai Irastorza-Azcarate, Rafael D. Acemel, Juan J. Tena, Ignacio Maeso, José Luis Gómez-Skarmeta, Damien P. Devos

**Affiliations:** Centro Andaluz de Biología del Desarrollo (CABD), Consejo Superior de Investigaciones Científicas/Universidad Pablo de Olavide, Seville, Spain; University of Pennsylvania, UNITED STATES

## Abstract

The use of 3C-based methods has revealed the importance of the 3D organization of the chromatin for key aspects of genome biology. However, the different caveats of the variants of 3C techniques have limited their scope and the range of scientific fields that could benefit from these approaches. To address these limitations, we present 4Cin, a method to generate 3D models and derive virtual Hi-C (vHi-C) heat maps of genomic loci based on 4C-seq or any kind of 4C-seq-like data, such as those derived from NG Capture-C. 3D genome organization is determined by integrative consideration of the spatial distances derived from as few as four 4C-seq experiments. The 3D models obtained from 4C-seq data, together with their associated vHi-C maps, allow the inference of all chromosomal contacts within a given genomic region, facilitating the identification of Topological Associating Domains (TAD) boundaries. Thus, 4Cin offers a much cheaper, accessible and versatile alternative to other available techniques while providing a comprehensive 3D topological profiling. By studying TAD modifications in genomic structural variants associated to disease phenotypes and performing cross-species evolutionary comparisons of 3D chromatin structures in a quantitative manner, we demonstrate the broad potential and novel range of applications of our method.

## Author summary

Chromatin conformation capture (3C) methods have revealed the importance of the 3D organization of the chromatin, which is key to understand many aspects of genome biology. But each of these methods have their own limitations. Here we present 4Cin, a software that generates 3D models of the chromatin from a small number of 4C-seq experiments, a 3C-based method that provides the frequency of contacts between one fragments and the genome (one vs all). These 3D models are used to infer all chromosomal contacts within a given genomic region (many vs many). The contact maps facilitate the identification of Topological Associating Domains boundaries. Our software offers a much cheaper, accessible and versatile alternative to other available techniques while providing a comprehensive 3D topological profiling. We applied our software to two different loci to study modifications in genomic structural variants associated to disease phenotypes and to compare the chromatin organization in two different species in a quantitative manner.

This is a *PLOS Computational Biology* Methods paper.

## Introduction

The three-dimensional (3D) architecture of the genome is important for most of its functions, such as gene expression regulation and DNA replication[1–3]. As with proteins, knowledge of the 3D structure of a genomic locus can reveal information not accessible from its primary sequence only. Indeed, the use of chromosome conformation capture (3C) methods together with high-throughput sequencing has profoundly changed our understanding of the 3D nuclear organization, adding a new dimension to the study of genome biology.

Amongst those new key findings is the discovery that the genomes of diverse animal lineages are organized in topologically associating domains (TADs)[4–7], genomic regions that typically span less than one Mbp within which the chromatin has a higher propensity to interact with itself. TADs are broadly preserved in interphase across different cells[4,8], they provide a structural basis to regulatory landscapes[1,9] and their structural perturbation has been linked to diseases[10–12]. Accordingly, TADs are largely conserved across different species[4,13,14].

Despite the growing interest in studying genomic information from a 3D perspective, 3C-based methods are still far from reaching their full potential to investigate a wider range of biological questions, partly because of the inherent limitations of these methods. All 3C technologies are based on similar biochemical principles to capture chromatin interactions, although with important variations (reviewed in [15,16]). They all start by cross-linking chromatin fragments that are located in close proximity in the nuclear space; the genome is then digested and ligated to capture interacting regions. Afterwards, these regions are identified and quantified by PCR or sequencing. Each 3C technique has its own experimental biases, but more importantly, they have different scopes, resolutions, costs, sequencing depths and data processing requirements[15]. Hi-C addresses chromatin contacts between all the regions in the genome and it is currently the only technique that allows the identification of genome-wide, large-scale genomic organizational features. However, this comes at the cost of losing power to determine fine-scale intra-TAD interactions, which are precisely the ones responsible for the regulation of individual genes and therefore of special interest in a variety of biomedical and genetic fields. This can in principle be overcome by performing Hi-C at the highest possible resolution, but this requires sequencing several billions reads per sample, implying financial costs exceedingly high for the vast majority of laboratories. 4C-seq (Circular Chromosome Conformation Capture) provides a good alternative solution for some of these problems. This technique is able to identify all the interactions of a given region of interest, usually termed ‘viewpoint’. With just ~1 million reads, 4C-seq can generate detailed high-resolution interaction profiles for a single locus. This high sensitivity and reduced sequencing cost has made this method particularly suitable for studies comparing multiple samples, between different species, genotypes or developmental stages, where it has been widely used to identify interactions between distal enhancers and gene promoters. Moreover, the recently developed NG Capture-C (next-generation Capture-C) technique[17] yields 4C-seq-like data in a high-throughput manner and of a higher resolution, making it a suitable technique to get detailed information of a certain locus, since multiple probes for multiple viewpoints within the region of interest can be designed.

Notwithstanding these advantages, both 4C-seq and NG Capture-C have also important limitations and provide incomplete information about TAD topology and borders, even when several viewpoints are used. Thus, in the absence of complementary Hi-C information from the same species, it may be difficult to get a complete and integrated picture of the interactions of a certain region. Finally, other technologies such as 5C (Chromosome Conformation Capture Carbon Copy) and Capture Hi-C (when designed to target a particular region using a tiled oligonucleotide capture approach), bridge somehow the gap between Hi-C and 4C-seq, being able to identify the large scale 3D chromatin organization of a given locus together with a high resolution contact map. Furthermore, as in the case of 4C-seq, they require a modest amount of sequencing depth. However, both approaches rely on the use of hundreds to thousands of probes or oligonucleotides from which the interaction profiles are identified and the costs and experimental design to produce these probes are far from trivial.

In sum, currently there is no experimental tool that combines, in a cost-effective manner, high-depth interaction profiles for particular loci with Hi-C-like information on TAD-level organization, hampering the accessibility of C-techniques to a wider number of scientists that will strongly benefit by incorporating 3D chromatin studies in their research.

Integrative modeling methods provide versatile approaches to infer 3D structures, since they are able to consider information derived from different techniques simultaneously. There are several integrative modeling method tools available at the moment that given a matrix of distances between genomic elements inferred from 3C contact frequencies, can compute the localization in the 3D space of these genomic elements[18–21]. These methods mostly use 5C or Hi-C based matrices as input data for the reconstruction of the genome structure, but none of them use 4C-seq-like data[22–25]. We have recently shown that 3D chromatin models can be successfully reconstructed from a small number of 4C-seq interaction profiles[3]. Here, we present 4Cin, a completely automated and easy to use pipeline to generate 3D chromatin models from 4C-seq data. 4Cin can also generate models using 4C-seq-like data coming from recently developed techniques such as NG Capture-C or Capture-C, as long as they are used to capture at least 4 viewpoints within each region of interest. 4Cin also allows the generation of vHi-C maps, the identification of TADs boundaries, the comparison of 3D structures and the integration of 3D structures with different epigenetic features. Here, we show the utility of 4Cin with two detailed case-studies that highlight some of the most important fields of application of our method: the study of genomic loci affected by structural variations causative of aberrant phenotypes using the mouse *Shh* locus, and evolutionary comparisons of 3D chromatin structures across different vertebrate species using the Six gene clusters.

## Results

### The tool: 4Cin, a 4C-seq to 3D pipeline

**Fig 1:**
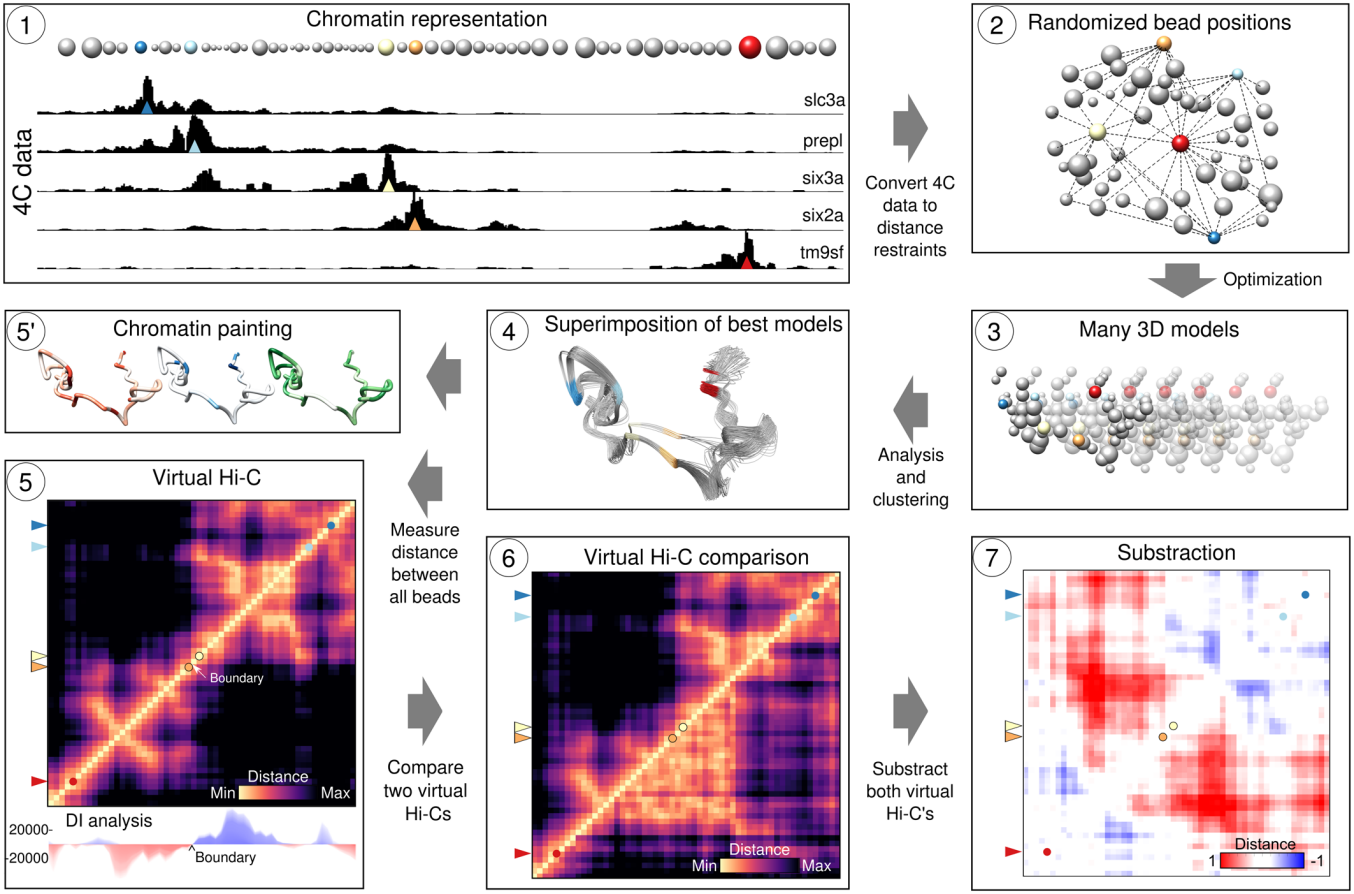
4Cin pipeline. (1) A genomic locus is represented as concatenated beads. Beads representing the viewpoints are color coded. The size of the beads is proportional to the size of their corresponding 4C-seq fragments. 4C-seq data is translated into distance restraints that are used in the optimization step. (2) Bead positions are optimized from random start positions. (3) Models that fulfill most of the restraints (i.e. with the best scores) are gathered and clustered based on their RMSD. (4) Models belonging to the most populated cluster are gathered and superimposed. (5') The most representative model can be painted using genomic or epigenomic data. (5) Distance between the beads representing the 3D models is measured from the population of best models and represented as a virtual Hi-C. Directionality index can be calculated to infer TAD boundaries. Two virtual Hi-C's can be compared (6) and subtracted (7).

4Cin was developed as an alternative to Hi-C to study particular genomic regions. Data from 4C-seq experiments are integrated to obtain 3D models that are represented afterwards as a vHi-C, a Hi-C like matrix of a given genomic locus (Fig 1 and S1 Fig). The tool was developed around IMP, the integrative modeling platform[26]. The tool was developed to handle data coming from multiple cells. Thus, the output models are representative of the average conformation of the chromatin in all cells and variability between models has not been shown to be related to chromatin dynamics.

#### Modeling the chromatin as a string of beads

The genome is represented as a flexible string of beads (Step 1 in Fig 1). The diameter of the beads corresponds to the theoretical length of the portion of straightened chromatin that we are representing, assuming the canonical chromatin width of 30 nm[27,28]. Beads are allowed to inter-penetrate, since we assume that the chromatin is unlikely to be straightened, occupying the full volume of the bead. We have previously shown that this type of representation generates robust results[3].

#### 3D reconstruction of the chromatin: 4C-seq counts as a proxy for distances

The central assumption of all 3C-derived integrative modeling methods is that read counts and physical distances are inversely related; high read counts connecting two DNA fragments imply close proximity between them whereas low counts imply larger distances. Accordingly, 4Cin uses these distance proxies as restraints (Steps 1 and 2 in Fig 1). Therefore, each 4C-seq experiment includes sequencing data that are interpreted as a pool of distances to the corresponding viewpoint. After various iterations of optimization of the position of the beads and evaluation of their fit with the restraints, a model that fulfills as many of the distance restraints as possible is generated. The optimization procedure combines a Monte Carlo exploration with steps of conjugate gradients as local optimization and simulated annealing. The fulfillment of the restraints is expressed as a score, where a score of 0 represents the fulfillment of all the restraints. The optimization process for each model ends when the score reaches a plateau or reaches 0. The process is repeated many times, generating many (typically 50000) models, in order to explore as completely as possible the variability between the models (Step 3 in Fig 1). A subset of the models that best fits the available data (i.e. those with the best scores) is analyzed afterwards (Step 4 in Fig 1). The end point of 5C or Hi-C experiments is a matrix of contact frequencies represented as a heat map. Hi-C heat map plots show the frequency of interaction between all pairs of DNA fragments which, given the initial 3C assumption, is used as a proxy for spatial proximity. A contact map mimicking a Hi-C heat map, in essence, a ‘vHi-C map’, can be generated by averaging the distances between all beads in the best 3D models (Step 5 in Fig 1).

To check the robustness of our method, we have generated 3D models of the *six2a-six3a* locus in zebrafish and generated vHi-Cs down-sampling the input 4C data, using a variable percentage of the original 4C-seq read counts to generate the models. The high correlation (Spearman rank correlation ρ > 0.7) of the vHi-C’s even when only 5% of the original data are used in the modeling, proves that 4Cin is robust to the sequencing depth of the underlying 4C data. We also carried out an unbalanced down-sampling, where three of the five 4C-seq experiments where down-sampled 95% and we also generated models where the raw 4C-seq data was modified, inserting read counts corresponding to the value representing the 95^th^ percentile of the data, as erroneous data, in randomized positions. We generated 3 rounds of modeling, with 1%, 2% and 5% of errors inserted. We were still able to get high correlations (Spearman rank correlation ρ > 0.7), supporting even more the robustness of our method (S2 Fig).

Our tool can be parallelized, allowing an acceleration of the process. 50.000 models based on a data set of 5 4C-seq experiments and represented by 56 beads, can be generated in about half an hour, on a computer with 20 cores and CPU’s of 2.5GHz. A region with 14 different 4C-seq experiments and 211 beads can be modeled in 7 hours.

#### Choosing the viewpoints

4Cin modeling is possible with as few as four 4C-seq datasets (distances from four different viewpoints; to be able to position each DNA fragment of the genomic locus in 3D space), but it is important to take into account that in order to leverage the complementarity of the data, the viewpoints should be well distributed along the entire locus. To show the importance of the distribution of the viewpoints, we modeled the *Six2-Six3* locus in mouse (Section 3.3) with three different sets of four viewpoints (S3 Fig). The correlation between the vHi-Cs and the original Hi-C suggests that a small number of viewpoints can generate reliable models, as long as these viewpoints are well distributed along the locus and not focused near the corners.

Importantly, we have previously shown with jackknifing experiments that vHi-C maps obtained from 3D models are very robust in terms of the number of viewpoints used, being able to accurately recapitulate original vHi-C results even when 10 out of 14 viewpoints are eliminated (average increase in correlation of 0.12)[3].

Therefore, although the quality of the 3D reconstruction improves by increasing the number of viewpoints provided, this improvement is relatively minor and furthermore, it is paralleled by an increase in computational cost. Thus, based on our experience[3], data coming from between four and ten 4C-seq assays are enough to achieve reliable models of a locus of 2Mbp.

The quality of the data is also important in order to generate reliable models. The tool provides a script to check the quality of the 4C-seq data before starting with the modeling steps (S4 Fig). Kurtosis and skewness values are calculated in order to check the suitability of the data for the modeling[29]: Kurtosis value measures the shape of the distribution, accounting for the central peak and the tails, while skewness value informs about the symmetry of this distribution.

#### Postprocessing analyses: TAD border calling, vHi-C comparisons and genome painting

TADs are major organizational elements of the chromatin and their organizations are informative about the overall architecture of specific loci. We provide a script that identifies TAD boundaries using the directionality index[4] (Step 5 in Fig 1). The script calculates the directionality index iteratively, ranging between the biggest (all beads) and smallest (one bead) possible size for a TAD, delivering a set of potential TAD boundaries.

TADs display important structural information, but combining 3D chromatin structure with epigenetic data can also reveal valuable information that is more difficult to observe from a linear perspective. Beads representing the chromatin can be colored with gradients according to genomic and epigenomic data. As examples, here, we colored the representative chromatin model of the wild type *Shh* locus using CTCF ChiP-seq data (GEO accession: GSM918741)[30]. As expected, the beads with the highest read counts are found near the TAD boundaries[8,14,31,32] and contain high score CTCF binding motifs. We also checked for the orientation of the CTCF binding sites in these peaks and observed the convergent orientation typically found flanking chromatin loops[8] (S5 Fig and S1 Table).

Moreover, two scripts to compare vHi-Cs are provided in the 4Cin package. One allows comparing the organization of homologous loci in different species providing conserved regions, which generates a heat map where each triangle represents a locus. The other one permits the comparison of different conformations of a region that underwent structural variation or mutation. This one yields a subtraction of both vHi-Cs. Both scripts calculate the correlation between the vHi-Cs that are being compared.

Below we demonstrate the use of the different tools implemented in our 4Cin method studying structural variations as well as evolutionary comparisons of 3D chromatin structures.

### Structural variation studies: Disruption of long-range regulation in the *Shh* locus

**Fig 2:**
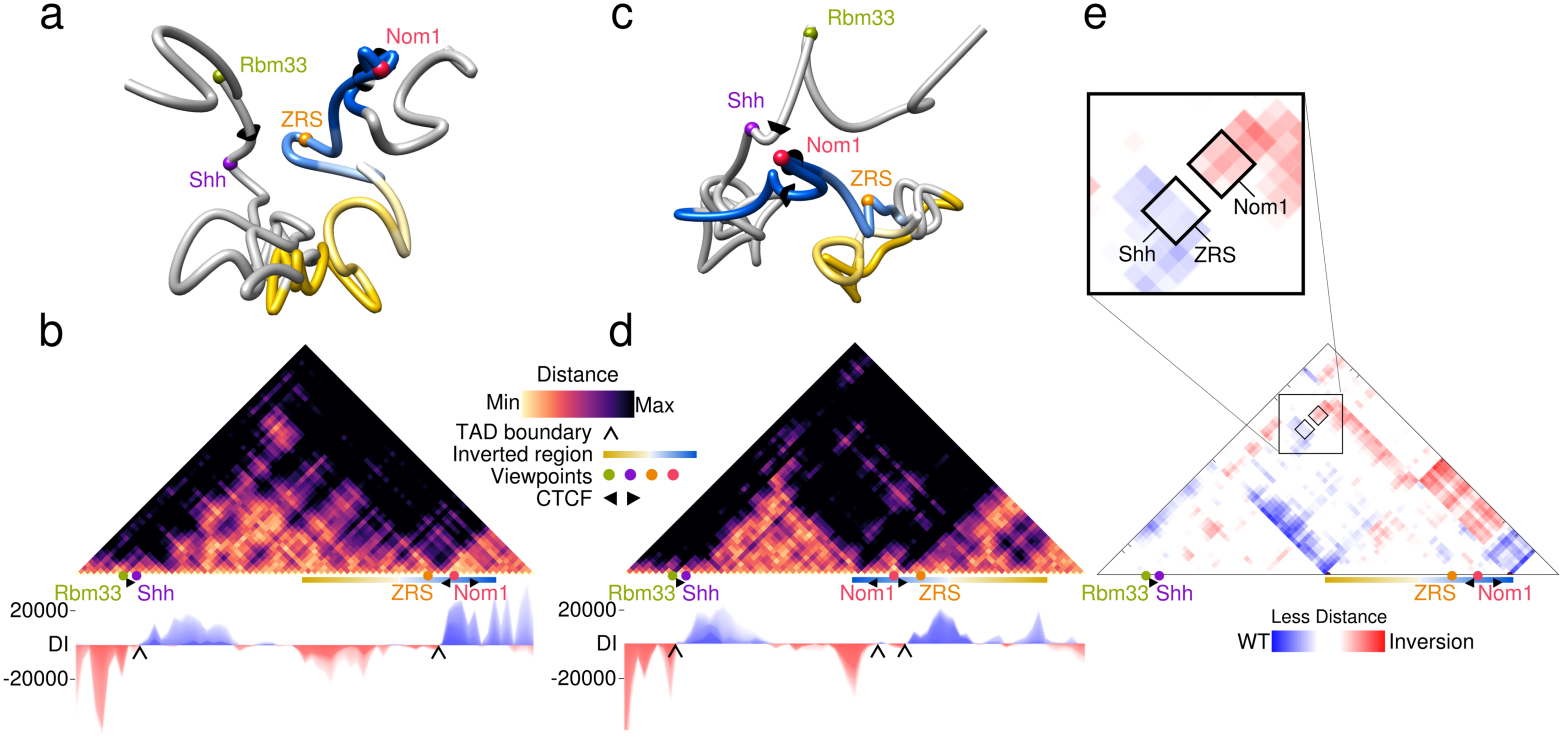
ZRS enhancer lies outside the *Shh*-TAD in mutant mice for the INV(6-C2) inversion. (a) Representative 3D model of the WT *Shh* region. Viewpoints are depicted as colored beads. CTCF binding sites are represented as oriented cones. The genomic region included in the inversion is colored with a yellow-to-blue gradient. (B) Virtual Hi-C of the WT *Shh* region (Top). Directionality index (Bottom) was applied to call TAD boundaries, showed with black arrows. (C, D) 3D model and virtual Hi-C heat map of the INV(6-C2) mutant, showed as in (A) and (B). (E) Subtraction of heat maps (B and D), blue corresponds to shorter distances in the WT, red to shorter distances in the mutant. The zoom-in shows that in WT mice, *Shh* is close to ZRS and far away from *Nom1* in comparison with the mutant.

Genomic mutations that compromise the structural integrity of TADs, such as inversions, duplications and boundary element deletions, have been shown to cause severe transcriptional mis-regulation of their associated genes, leading to the appearance of diverse disease phenotypes[10–12]. To illustrate the utility of 4Cin in understanding the molecular nature and effects of these structural genomic mutations, we focused on the region surrounding the gene sonic hedgehog (*Shh)*, a locus encoding a key diffusible signaling molecule for vertebrate development. *Shh* regulatory landscape spans over 900 kb, comprising several unrelated neighboring genes and multiple long-range enhancers, including one of the most distal enhancers identified so far, the *Shh* limb-specific enhancer known as ZRS. Previous works using 4C-seq data have shown that in mice with genomic mutations in the *Shh*-TAD, such as inversions, deletions and duplications, *Shh* regulatory interactions and expression were impaired, causing severe malformations[33]. In particular, INV(6-C2), a large 600 kb inversion encompassing nearly half of the *Shh*-ZRS TAD, greatly diminished 4C-seq contact frequencies between the ZRS and the *Shh* promoter. By applying 4Cin to these published 4C-seq datasets, we generated 3D models for both the wt *Shh* locus and the INV(6-C2) inversion mutant genotype (Fig 2).

This revealed that the two corresponding chromatin topologies are markedly different: whereas in the wt *Shh* and the ZRS lie in close proximity, they are widely separated in the inversion (Fig 2A and 2C). In fact, vHi-C maps derived from these models and subsequent TAD border calling showed that the inversion completely changed the relative locations of some of the TAD boundaries, most likely due to changes in the relative orientations of the CTCF binding sites located next to *Nom1* and ZRS (Fig 2B and 2D and S5 Fig).

Thus, in the mutant genotype, the ZRS enhancer together with nearly half of the *Shh* regulatory landscape, are now part of another TAD. This enhancer is therefore isolated from the *Shh* promoter, explaining the reduced contact frequencies observed previously[33]. Indeed, a global quantification of distance changes across the entire locus by comparing the two vHi-Cs contact matrices showed that the distance between ZRS and *Shh* in the 3D models increases in the inversion (Fig 2E).

#### The topology of the *Shh* locus explains its regulatory organization

**Fig 3:**
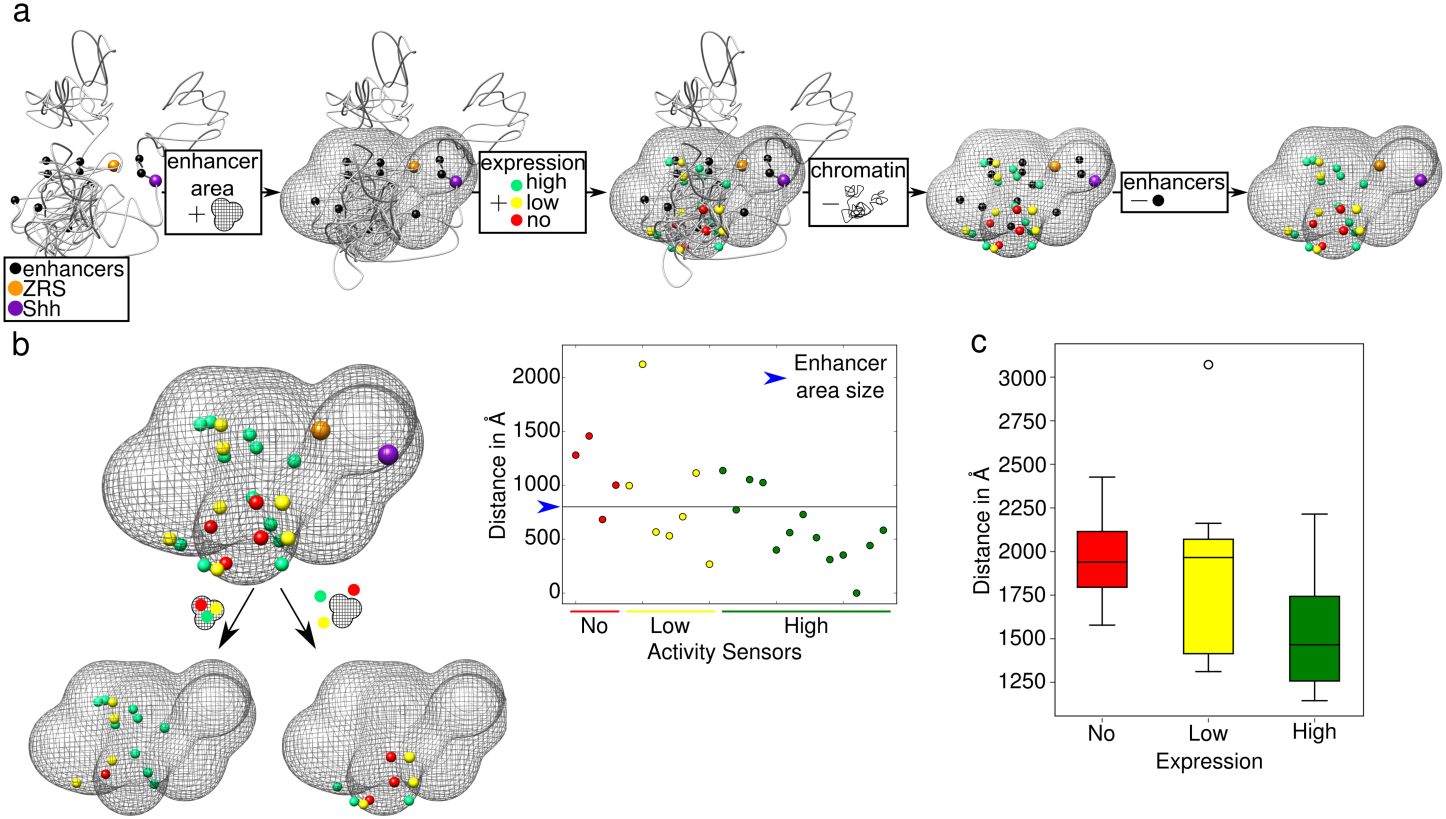
Insertion sensors with high responsiveness are close to enhancers in the 3D space. (A) Stepwise explanation showing how we obtain top figure in panel B. Beads are color coded to indicate different regions: enhancers (black), *Shh* promoter (purple), ZRS (orange), and the three type of insertions, high, low and no expression (green, yellow and red, respectively). The enhancer area at 75 nm away from the enhancers is shown with a gridded surface. (B) Insertions, *Shh* and ZRS locations relative to the enhancer area. Below, beads that are outside and inside the area are depicted. On the right, plot showing the distance between enhancers and insertions. (C) Boxplot showing average distance between beads representing the insertions and enhancers.

Using a large collection of insertions of regulatory sensors at multiple locations within the *Shh* regulatory landscape, the responsiveness to enhancers of different regions within the *Shh*-ZRS TAD was evaluated[33,34]. The results showed that most regions within the TAD were able to respond to at least some of the multiple tissue-specific *Shh* enhancers. However, there were a few insertion locations with no or very little responsiveness. Given that these regulatory “blind spots” did not show any particular location trend in terms of their linear distance to the enhancers (in particular to the ZRS) or local chromatin features such as histone marks or accessibility, the authors hypothesized that the lack of responsiveness may be related to their position within the 3D native structural folding of the locus. To test this hypothesis we mapped the positions of all the insertion sensors to a high resolution 3D chromatin model. We also located the positions of the comprehensive collection of *Shh* regulatory elements so far identified[35–40], which allowed us to define a 3D space containing all known *Shh* enhancers (Fig 3A). We then classified insertion sensors into three groups (high, low and no expression) depending on the level of expression of their associated reporter genes[33,34,41] (S2 Table). Consistent with the proposed hypothesis[33], these different expression activities of the sensors correlated inversely with their average distance to the enhancers (Spearman rank correlation ρ < 0.05) (Fig 3C), accordingly, most of the high expression sensors fell inside the enhancer area (Fig 3B). This supports the idea that the low enhancer responsiveness of certain chromatin regions is related to their topological position and their ability to interact with the different enhancers in the locus.

In conclusion, in comparison with 4C-seq alone and without generating any additional experimental data, the use of 4Cin provides further and deeper insights into the structure and regulatory interactions of a chromatin locus, generating a more complete characterization of the region, with identification of TAD borders, quantitative comparisons between different genetic backgrounds and testing specific hypothesis related to topological interactions.

### Comparative genomics and evolution: Conserved 3D chromatin structures in the *Six2-Six3* gene cluster of bony vertebrates

The evolution of genome architecture in animals has been traditionally studied using comparative genomics methods that can only consider DNA sequences from a linear perspective. The advent of 3C-based techniques has literally added a new dimension to this field, but so far cross-species comparisons of 3D chromatin structures have been performed only in a handful of species (in particular mammals) and have mostly relied on the use of Hi-C data[4,13]. This situation currently restricts the development of 3D-aware comparative genomic studies, since they would ideally involve the use of evolutionary relevant species for which Hi-C data is either still unavailable or difficult to produce, especially in cases comparing multiple lineages. We applied 4Cin to compare orthologous genomic loci, the *Six2-Six3* gene clusters, from two bony vertebrate species, mouse, a mammal with several published Hi-C data, and zebrafish, a teleost fish for which Hi-C data are still unavailable.

#### The Six2-Six3 locus is conserved in vertebrates

Six homeobox genes are essential developmental regulators organized in genomic clusters conserved in multiple animal phyla[14,42]. The clusters consist of three subfamilies: Six1/2, Six3/6 and Six4/5. Due to the two rounds of whole genome duplications that happened at the origin of vertebrates, most species within this group have two paralogous copies of the cluster, one containing *Six2* and *Six3* genes, and the other containing *Six1*, *Six6* and *Six4* genes. Teleosts, like zebrafish, have undergone another round of duplication and contain four Six clusters.

**Fig 4:**
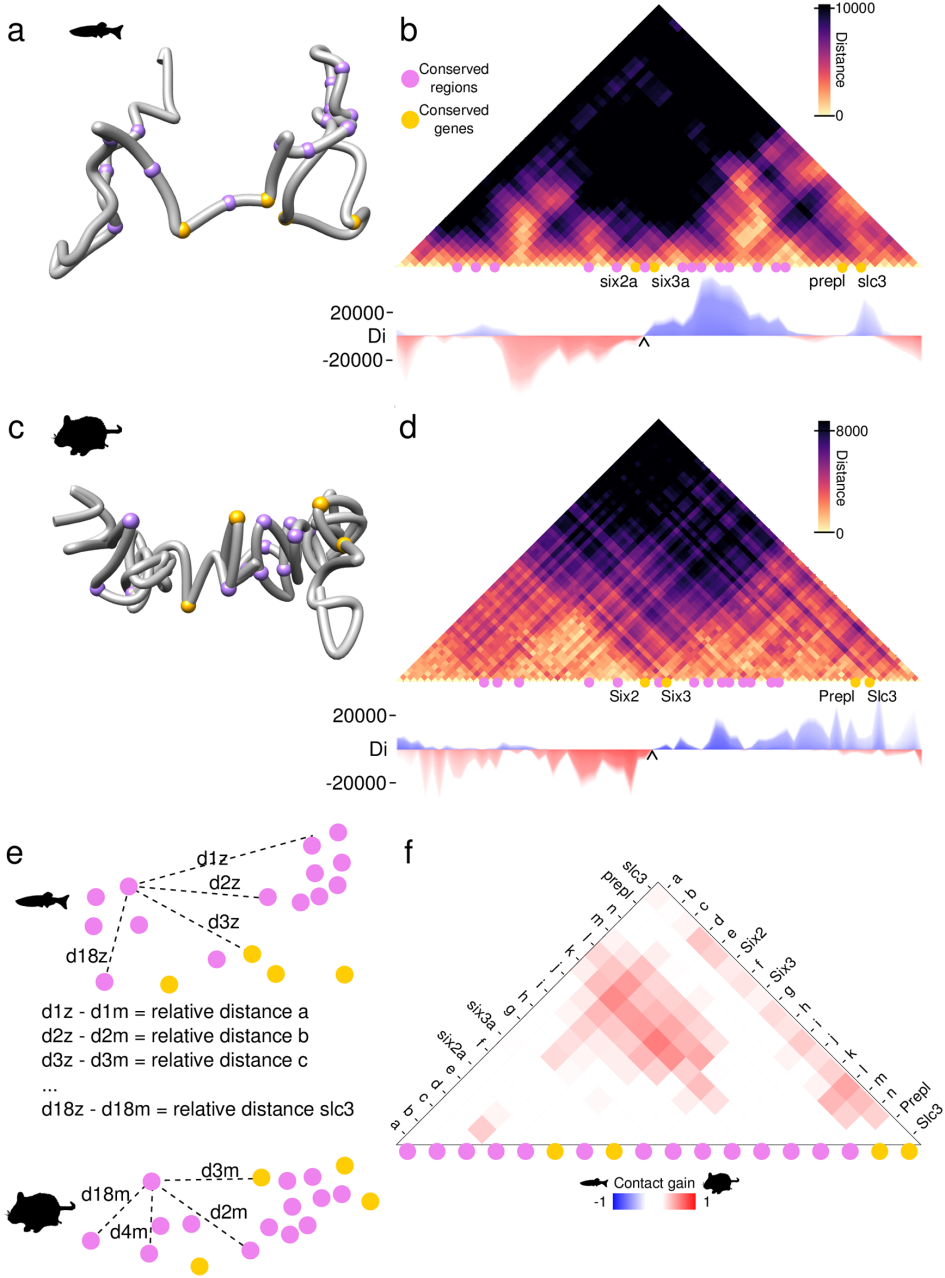
Mouse and zebrafish *Six2-Six3* clusters have conserved 3D topologies. (A) Representative 3D model of the *six2a-six3a* gene cluster in zebrafish. Orthologous regions conserved at the sequence level between the two species are depicted as purple beads, genes are indicated with yellow beads. (B) Virtual Hi-C of the zebrafish *six2a-six3a* cluster. Directionality index shows the TAD boundary, represented by a black arrow. (C, D) Representative 3D model of the same cluster and the Virtual Hi-C from mouse, labeled as in (A, B). (E) Changes in relative distances between conserved regions are obtained by subtracting the relative distance values of the two species. (F) Subtraction heat map of the distance changes obtained from (E). Red squares indicate shorter distances in mouse, blue squares indicate shorter distances in zebrafish.

Here we use available 4C-seq data to explore the conformation of the cluster containing the *six2a* and *six3a* genes in zebrafish, which has been described to have a bipartite organization that split the regulatory landscapes of each of these genes into two different adjacent TADs[14]. The 3D models of the *six2a-six3a* locus in zebrafish and their derived vHi-C show two TADs with the Six genes located between them (Fig 4A and 4B), corroborating previous results based on 4C-seq profiles only[14].

We also generated 3D models of the mouse *Six2-Six3* locus using publicly available mouse Hi-C[4] converted into virtual 4C-seq like data (Fig 4C and 4D). From those 3D models, we derived a vHi-C that shows high correlation with the real Hi-C (Spearman rank correlation ρ = 0.86, S3B Fig), that provides further support to our method, in agreement with our previous observations[3].

In order to quantify the degree of structural similarity of mouse and zebrafish *Six2-Six3* clusters, we focused on a set of 18 regions that are conserved at the sequence level between the two species, comparing the distance heat maps corresponding to these regions (Fig 4E and 4F and S3 Table). The strong correlation observed between these two sets of distances (Spearman rank correlation ρ = 0.81) shows the high degree of topological conservation in the two species. Indeed, the two species have maintained very similar relative distances between these conserved regions, with an average change of just 20% (S6 Fig). Interestingly, the vast majority of distance changes were all in the same direction, decreasing their relative distances in mouse in comparison with zebrafish (red bins, Fig 4F). We hypothesize that the greater compaction in mouse helps compensate for the larger sequence length of this species, maintaining therefore similar 3D structural organizations in the two vertebrate lineages. Nevertheless, we are aware that the differences in the techniques used to model both loci could influence the final modeling.

Directionality index analysis[4] was also applied in these regions to call TAD boundaries (Fig 4B and 4D). A TAD boundary is found between the genes *Six2* and *Six3* in both species, supporting the conserved bipartite configuration of the clusters. Thus, our results show that the evolutionary conservation of gene expression in this cluster is not only due to the presence of conserved regulatory regions but also to a largely constrained 3D chromatin topology along the vertebrate lineage.

## Discussion

Thinking about the chromatin as a 3D structure and trying to unravel its spatial organization have become necessary steps to properly understand genetic information in a functionally coherent manner. 3C-based methods can help to achieve this goal, but existing techniques provide different compromises between resolution, scope and costs and can therefore be difficult to implement from economical and technical points of view.

The tool presented here, 4Cin, can generate 3D models and derive vHi-C contact maps from a reduced number of 4C-seq datasets, uniting some of the specific advantages of different 3C techniques in a cost-effective manner. This makes 4Cin particularly useful for a broad range of single-locus studies dealing with multiple samples, conditions or species in which detailed 3D chromatin profiling was until now economically unfeasible. We have illustrated this with detailed examples showing the important biological implications and the multiple possibilities to test specific hypotheses that 4Cin can offer.

In order to generate reliable 3D models, various steps of the process have to be taken with additional caution: Data obtained from different species, tissues, time points or different experiments (like simulating virtual 4C-seq data from Hi-C data) should be carefully harmonized before integration with 4Cin, and, likewise, a proper normalization of these data has to be carried out (check 4C-seq data processing in Methods section). In addition, the tool expects data to be derived from multiple cells and it is not optimized to be used with single-cell 3C-based experiments.

We believe that 4Cin will expand even further the use, interest and applications of chromatin capture techniques, helping a growing number of researchers to switch the way in which genomic information has been traditionally studied and generating new ideas, hypotheses and methods.

## Methods

In this work, we refined and automated our previous algorithm[3] and provide novel scripts to ease the postprocessing analyses of the results and the discovery of biological novelties. This tool generates 3D chromatin models from 4C-seq data. The code is public and available at https://github.com/batxes/4Cin with a GNU GENERAL PUBLIC LICENSE. The usage of the pipeline (Fig 1 and S1 Fig) is also explained in the repository link. The 4Cin pipeline can be deployed pulling the docker image from https://hub.docker.com/r/batxes/4cin_ubuntu/ to avoid the installation of the dependencies. The input data and the final 3D models of all the regions studied in this work are also uploaded in github.

Our method uses the Integrative Modeling Platform (IMP)[26] and is based on a previous work[22]. The 3D models are composed of beads representing chromatin fragments and 4C-seq data is encoded as distance restraints between these beads. IMP tries to fulfill these restraints that are expressed in a single scoring function that the optimization algorithm attempts to minimize.

### Chromatin representation

The chromatin is represented as a flexible chain of beads, each bead representing a fixed number of consecutive DNA fragments, as previously described[3]. In the *six2a-six3a* locus in zebrafish, 33 DNA fragments are represented as one bead, while for the same region in mouse, each fragment corresponds to one bead, depending on the data resolution. Each bead comprising the Shh locus in mouse, both wild type and the inversion mutant, represents 100 fragments. The size of these beads is proportional to the length of the represented fragments. Assuming a canonical chromatin width of 30 nm (6–7 nucleosomes per 11 nm fiber length[27,28]), the radius (r_i_) of these beads is defined as:

$${\text{r}}_{\text{i}}=0{.0423*\text{l}}_{\text{i}}$$

where l_i_ is the length of the DNA fragments represented in each bead. Our *Six2-Six3* loci models in zebrafish and mouse are represented with 56 and 75 beads, that, at the same time, are representing a region of 1,12 and 1,48 Mbp. The Shh locus is 1,41 Mbp long and is represented by 71 beads.

### 4C-seq data processing

4C-seq data were analyzed as previously described[43]. Briefly, raw sequencing data were demultiplexed and aligned using mouse July 2007 assembly (mm9) or zebrafish July 2010 (danRer7) as the reference genomes using bowtie[44]. Reads located in fragments flanked by two restriction sites of the same enzyme, or in fragments smaller than 40 bp were filtered out. Mapped reads were then converted to reads-per-first-enzyme-fragment-end units, and smoothed using a 30 fragment mean running window algorithm. To be more consistent, the 4C-seq data corresponding to the Shh region was processed as in Symmons et. Al[33]. For the INV(6-C2) genotype, we mapped the 4C-seq data and did all the subsequent analyses using a custom version of the mouse genome that incorporates the corresponding genomic inversion at the previously described breakpoints[33].

### 4C-seq data normalization

4C-seq data consists of frequencies of interactions between the viewpoint DNA fragment and the rest of the locus. Our modeling protocol is based on trilateration, so we need at least four distances to locate a bead in 3D space: due to the fact that the 4C-seq method provides information between a DNA fragment and the rest of the fragments, we need at least four 4C-seq experiments to determine the position of a fragment. Each 4C-seq experiment is done in different population of cells and, therefore, the output of each experiment is likely to vary in the number of read counts. Hence, we first adjusted the measured values of each experiment to the same scale, multiplying each read count in each 4C-seq experiment by a factor so that we get the same read counts as the 4C-seq experiment with the biggest number of read counts. For the *Six2-Six3* locus, we used 5 experiments in zebrafish and 10 in mouse, while the Shh locus was modeled with four 4C-seq experiments in both the wild type and the inversion (S4 Table). Afterward, a Z-score is assigned to each bead. The Z-score indicates how many standard deviations separates a datum from the mean, identifying pair of (sets of) fragments (in this case, pair of beads) that interacts more or less than the average interaction frequency. To calculate the Z-score, the data needs to follow a normal distribution. The 4C-seq data does not follow a normal distribution, therefore, read counts are transformed by applying a log_10_ transformation to achieve a normally distributed data[22]. The Z-score is computed as follows:

The standard score of a raw score *x* is

$$z=\frac{x-\mu }{\sigma }$$

where *μ* is the mean of the population and *σ* is the standard deviation of the population.

We set two thresholds called upper bound Z-score and lower bound Z-score. Contact frequencies that fall between both cutoffs are not used for the modeling as those interaction counts are more likely to happen by chance, since they don’t fall in the tails of the normal distribution (S4 Fig). The optimal values for these thresholds were calculated empirically (see **Empirical determination of upper and lower Z-scores**).

### Restraints and scoring function

As the chromatin fragments that they represent, consecutive beads were imposed to be connected by the application of harmonic upper bound distance restraints between consecutive beads. These distances are the sum of the radii of both consecutive beads.

We defined the “reach window” as previously described[3]. Briefly, the “reach window” of a 4C-seq experiment is the area between the furthest upstream and downstream fragments with a Z-score above the upper Z-score. Harmonic distance restraints were applied between beads corresponding to the viewpoints and the rest of the beads that were inside the reach window, as long as the Z-scores of the beads were not between the upper and lower Z-scores. Beads outside the reach window were restrained with harmonic lower bound distances. We set as weights the absolute values of the Z-scores of each bead, to give more importance to the beads with lowest and highest read counts.

The conversion from the read counts to the distance restraints is achieved by a linear relationship based on two assumptions: (I) the bead(s) with the maximum number of reads in each experiment will be imposed a harmonic distance restrain of 30 nm[27,28], (ii) the bead(s) with the minimum number of reads or zero reads, will be imposed a harmonic lower bound distance restrain equal to the maximum distance variable (see **Empirical determination of scale** and S4 Fig).

The sum of these restraints (Table 1) is the scoring function, a function that is minimized in each iteration. A scoring function of zero, means that all restraints are fulfilled, thus, this score represents the degree of consistency between the restraints and each 3D structure.

**Table 1:** Information on restraints.

Name	Restraint type	Functional form	distance(nm)	Bead i	weight(k)
U_conn_	Chromatin connectivity restraint	Harmonic upper bound distance restraint	R_i_+R_i+1_	i = {1..N-1}	1
U_irea_	Inside reach window chromatin restraint	Harmonic distance restraint	d_irea_	Any i ∊ β	|z-score|
U_orea_	outside reach window chromatin restraint	Harmonic lower bound distance restraint	d_orea_	Any i ∊ α	|z-score|

The IMP scoring function is defined as:

$$\text{S}\left({\text{r}}_{\text{i}},..,{\text{r}}_{\text{N}}\right)=\sum_{\text{i}=1}^{\text{N}-1}{\text{U}}_{\text{conn}}\left(r_{i},r_{i+1}\right)+\sum_{j=1}^{M}\left(\sum_{i\in \alpha }U_{irea}\left(r_{i},r_{j}\right)+\sum_{i\in \beta }U_{orea}\left(r_{i},r_{j}\right)\right)$$

r_i_ represents the 3D coordinate vector of each bead i, N is the total number of beads in the model, M is the list of assigned numbers to the viewpoint beads, α is the set of beads inside the reach window and β is the list of beads outside the reach window.

Chromatin connectivity restraints (U_conn_) restrict the beads to be connected within a distance which is the sum of the radii of both consecutive beads with harmonic upper bound distances.

Inside reach window chromatin restraints (U_irea_) impose harmonic distance restraints between beads inside this window and outside reach window chromatin restraints (U_orea_) impose harmonic lower bound distances between the rest of beads.

### Empirical determination of scale

Beads containing the lowest number of reads in each experiment will be located at the maximum distance away from the viewpoint. This distance in calculated empirically as follows. Models are generated varying this maximum distance in steps of 1000 (by default) and keeping the upper and lower Z-scores low, in order to take into account most of the distance restraints. Afterwards, the mean length of the models is calculated, summing the distance between consecutive beads in each model. Then, the theoretical length of the chromatin of the region we are modeling is calculated assigning a theoretical length of 0.846nm to each of the nucleotides[27,28]. The maximum distance of the models with a length closer to the theoretical optimum will be set for the final modeling. In the mouse and zebrafish six loci, 11000Å and 13000Å were set as maximum distances. On the other hand, the maximum distance in the Shh locus was of 10000Å for the wild type and 7000Å for the inverted region.

### Empirical determination of upper and lower Z-scores

A similar approach as in “Empirical determination of scale” is used to set the upper and lower Z-score parameters. In this case, models are generated with the previously obtained maximum distance fixed, varying the upper and lower Z-score parameters, in bins of 0.1 by default. Then, the distance between the viewpoint beads and the rest of the beads is measured and the mean of these distances is obtained from all the generated models to compare with the raw 4C-seq data (S7 Fig). The Z-scores of the set of models that correlate best with the raw data is used in the final modeling. For the zebrafish six locus 0,1 and -0,1 were set as the uZ and lZ, while 0,2 and -0,1 were set for mouse in the same region. Likewise, 0,1 and -0,1 was set in the Shh region for the wild type, and 0.2 and -0.1 were the values for the inverted region.

### Optimization

With the maximum distance and upper and lower Z-scores fixed, models are generated starting from entirely random set of positions of the beads. The number of models should be big enough in order to sample the space of solutions thoroughly and allow a reliable analysis afterwards. In this work, 50.000 models were generated in all 4 examples. We have seen that generating less than 10.000 models can lead to very variable models (S8 Fig). The modeling is carried out with IMP, optimizing the scoring function. The algorithm combines a Monte Carlo exploration with steps of local optimization and simulated annealing. The optimization ends when the score difference between the rounds is below 0.00001 or when the score reaches 0.

### Analysis and clustering of models

From the whole population of models, the models with the best score are selected as long as they fulfill most of the distance restraints. The standard deviation and the percentage of restraints fulfillment is used to filter out unreliable models. The method starts from a very low distance and high restraints fulfillment percentage and does many analysis iterations loosening up these cut-offs until 200 models can be retrieved. We selected the 200 models with best score as long as they fulfilled 85% of the restraints for zebrafish and mouse, with an std-dev of 2000Å and 2250Å as a limit of restraint fulfillment. We also selected 200 models with best score for both wild type and inverted Shh locus that fulfilled 85% of the restraints with an std-dev of 1000Å and 960Å respectively.

Then, these models are clustered according to their similarity measured by the Root Mean Square Deviation (RMSD). The goal of this step is to identify mirror image models since we don't have information to discriminate the mirror images. The set of models for both zebrafish and mouse and the wild type and inverted Shh locus were clustered showing two mirror image clusters (S7 Fig).

The number of clusters depends on: 1) the quality of the modeling, but also 2) the structural variability of the genome locus. The high number of clusters could indicate high structural variability, meaning the there is no enough data to filter between them or that the quality of the data is not good.

### Representative and superposition of models

4Cin selects the biggest cluster of models for next analyses. From the models in the selected cluster, the most similar model to the average of all the models is used as the representative model (Fig 3A). The superposition of all the set of final models is shown also to see the variability between them (S9 Fig). In addition, the variability of the beads between the models in the biggest cluster is shown.

### Virtual Hi-C generation and comparisons

A contact map is generated resembling a Hi-C heat map plot, that we called virtual Hi-C (vHi-C). For this, the average distance between pair of beads from the best models is calculated (Figs 2B and 3B). vHi-C’s of the wild type and inverted Shh region were compared and a subtraction of both virtual Hi-C’s was done, repositioning the inverted region as in the wild type, in order to compare the change in contacts of each bead. The heat map shows in blue the contacts that were lost in the inversion, and in red the contacts that were gained (Fig 2C). To compare the six loci in both species quantitatively, we measured the distance between beads that represent conserved regions in both mouse and zebrafish (Fig 4, S3 Table). In our models, each bead represents almost the same amount of nucleotides, 20Kbp, making the comparison of conserved regions more reliable.

### Generation of the 4C-seq mouse data form Hi-C data

The virtual 4C-seq data representing the viewpoints containing the *six2* and *six3* genes and the data of 8 other scattered viewpoints was extracted from the original Hi-C data from Dixon et. al.[4] (S4 Table). This data was used as 10 single 4C-seq experiments to generate the 3D models and the virtual Hi-C heat map plot.

### Directionality index

Directionality index (DI) was calculated in all the vHi-Cs as in ref 3 with slight changes. The DI for the beads at the edges of the vHi-Cs, was calculated by assigning the mean of all the values in the vHi-C for the heat map squares that are not represented in the vHi-C. We also calculated the DI iteratively, ranging the TAD size from 1 (size of TAD = 1 bead) to the total number of beads of the model (size of TAD = N). Afterwards, we overlaped all the DIs to generate the plot (Step 5 in Figs 1, 2B, 2D, 4B and 4D) and give a list of all the TAD boundaries called, sorted by the number of times that they were called in each iteration.

### Genome painting

CTCF Chip-seq data used in the Shh region was adquired from Encode https://www.encodeproject.org/experiments/ENCSR000CEB/ and painted in the representative model (mm9 data) with a black-to-white gradient, from high to low score.

### 3D models manipulation and surface calculation around enhancers

4Cin generates 3D models that can be opened and modified in UCSF Chimera (https://www.cgl.ucsf.edu/chimera/). The molmap command of UCSF Chimera was used to generate a mesh surface of 75 nm in radius around the Shh region enhancers in Fig 3.

### Determination of conserved regions between zebrafish and mouse

To define genomic regions conserved at the sequence level between mouse and zebrafish *Six2-Six3* clusters, we downloaded the corresponding chained alignments available in UCSC. We then manually inspected and curated these aligned regions to verify that their locations and orientations were equivalent in the two species and that they corresponded to bona-fide conserved sequences. Genomic coordinates are provided in S3 Table.

### CTCF directionality calculation

Clover (https://zlab.bu.edu/clover/) was used in the Shh region to predict CTCF binding sites and their orientation (S1 Table), using a mouse CTCF position weight matrix (http://cisbp.ccbr.utoronto.ca/TFreport.php?searchTF=T049038_1.02) and setting a threshold of 7.

### Mapping of insertion sensors and generation of the enhancer contact area

To map these positions with high accuracy, we generated 3D models of the Shh locus with a fivefold higher resolution. We then selected the best models and used the most representative model to map the enhancers and insertion sensors. The measurements between the enhancers and the sensors were carried out taking into account the whole population of best 3D models.

### 4C-seq data down-sampling and erroneous data insertion

Bed files that were mapped in the zebrafish genome (danrer10) were shuffled and then, the first 20%, 40%, 60%, 80%, 90%, and 95% lines of the files were removed to get the down-sampled data. Afterwards, the same procedure as in **4C-seq data processing** was followed. To generate a set of erroneous data, we calculated the value representing the percentile 95 of the data for each experiment, and switched with random read counts of pair of fragments.

## Supporting information

S1 Fig Schematic explanation of 4Cin. (Pipeline) First, the maximum distance, the upper bound Z-score (uZ) and the lower bound Z-score (lZ) are calculated so these parameters are afterwards used in the final modeling. Then, these models are subjected to an analysis to retrieve the best models and clustered based on their RMSD to distinguish between mirror image models. Best models can also be super imposed, to see structural variability. The representative model can be colored depending on genetic or epigenetic data. Finally, best models are used to generate a virtual Hi-C (vHi-C). Additionally, TAD boundaries can be called in the vHi-Cs and other vHi-Cs can be compared using the scripts provided with the pipeline. (Modeling) The modeling process first encodes the 4C-seq data into restraints. Distance restraints are also used to connect beads. These restraints and the representation of the chromatin fragments as beads are taken into account in the optimization process to generate a single model.
(PDF)

S2 Fig Correlation with down-sampled and erroneous 4C data. Pearson's and Spearman's correlation between the vHi-C derived from six2a-six3a zebrafish locus models and the vHi-C's of the same locus down-sampling and inserting errors in the 4C data.
(PDF)

S3 Fig Mouse Six2-Six3 cluster topology comparison. (a) Hi-C of the Six2-Six3 cluster (Gene Expression Omnibus (GEO) accession number GSM862722). (b) vHi-C of the Six2-Six3 cluster. (c,d,e) vHi-Cs of the Six2-Six3 cluster generated with different viewpoints. (f) Spearman's correlation between the Hi-C (a) and the vHi-Cs (b,c,d,e).
(PDF)

S4 Fig Output plot generated by the data_manager.py script. Example corresponds to the Six3 viewpoint in mouse. Top, 4C-seq read counts by bead. Red bar shows the viewpoint. Middle, Z-scores in red corresponding to the read counts from the top panel. Horizontal blue lines indicate the upper bound Z-score and lower bound Z-score. Bottom, Distance restraints encoded from the read counts in the top panel.
(PDF)

S5 Fig Shh-TAD boundaries are enriched in CTCF sites. (a) vHi-C of the Shh WT region on top. CTCF Chip-seq data corresponding to the region colored in white-to-black gradient, white for low reads, black for high reads. CTCF sites with highest reads are depicted with oriented triangles. (b) Shh WT representative model colored as in panel (a). Yellow beads represent Shh-TAD boundaries. Shh-TAD is encircled in yellow-black. (c and d) vHi-C, CTCF Chip-seq data and representative model depicted as in (a) and (b).
(PDF)

S6 Fig Zebrafish and mouse topology comparison. Subtraction heat map of the distance changes between conserved regions in the Six2-Six3 cluster in zebrafish and mouse as explained in Fig 2E. Top triangle corresponds to zebrafish data and bottom triangle to mouse data. Red squares indicate shorter distances in mouse, blue shorter distances in zebrafish.
(PDF)

S7 Fig Analysis and clustering of 3D models. (a, c, e, g) Heat maps comparing the raw 4C-seq data and the mean distances between beads of the models with the best parameters (upper bound Z-score, lower bound Z-score and max distance): Shh WT region: 0.1, -0.1, 11000; Shh inverted region: 0.2, -0.1, 10000; Six2-Six3 cluster in zebrafish: 0.1, -0.1, 13000 and in mouse: 0.2, -0.1, 11000. (b, d, f, h) Heat maps showing 2 clusters in the Shh WT and inverted region and the Six2-Six3 cluster in zebrafish and mouse. The clustering was performed based on the RMSD of the 3D models.
(PDF)

S8 Fig Variability depending on number of models. Average variability of the 3D models depending on the sampling.
(PDF)

S9 Fig Superposition of 3D models and their variability. Superposition of 3D models of the biggest cluster after clustering the best models of the Shh WT region and variability of each bead in the cluster showed in standard deviation devided by their maximum distance to show them at scale. (a), Shh inverted region (b), Six2-Six3 cluster in zebrafish (c) and mouse (d).
(PDF)

S1 Table Viewpoints used. Viewpoints used in the generation of 3D models.
(PDF)

S2 Table Conserved regions in Six2-Six3. Conserved regions and genes between Zebrafish and Mouse in the Six2-Six3 region.
(PDF)

S3 Table CTCF locations. Location and sign of CTCF binding sites.
(PDF)

S4 Table Sensor probes and enhancers. Sensor probes and enhancers in the Shh region.
(PDF)
