# Supplementary figures and images for: 4Cin: A computational pipeline for 3D genome modeling and virtual Hi-C analyses from 4C data
Source: PLoS Comput Biol. 2018 Mar 9;14(3):e1006030. doi: 10.1371/journal.pcbi.1006030 (PMC5862518; doi:10.1371/journal.pcbi.1006030)

## Pipeline

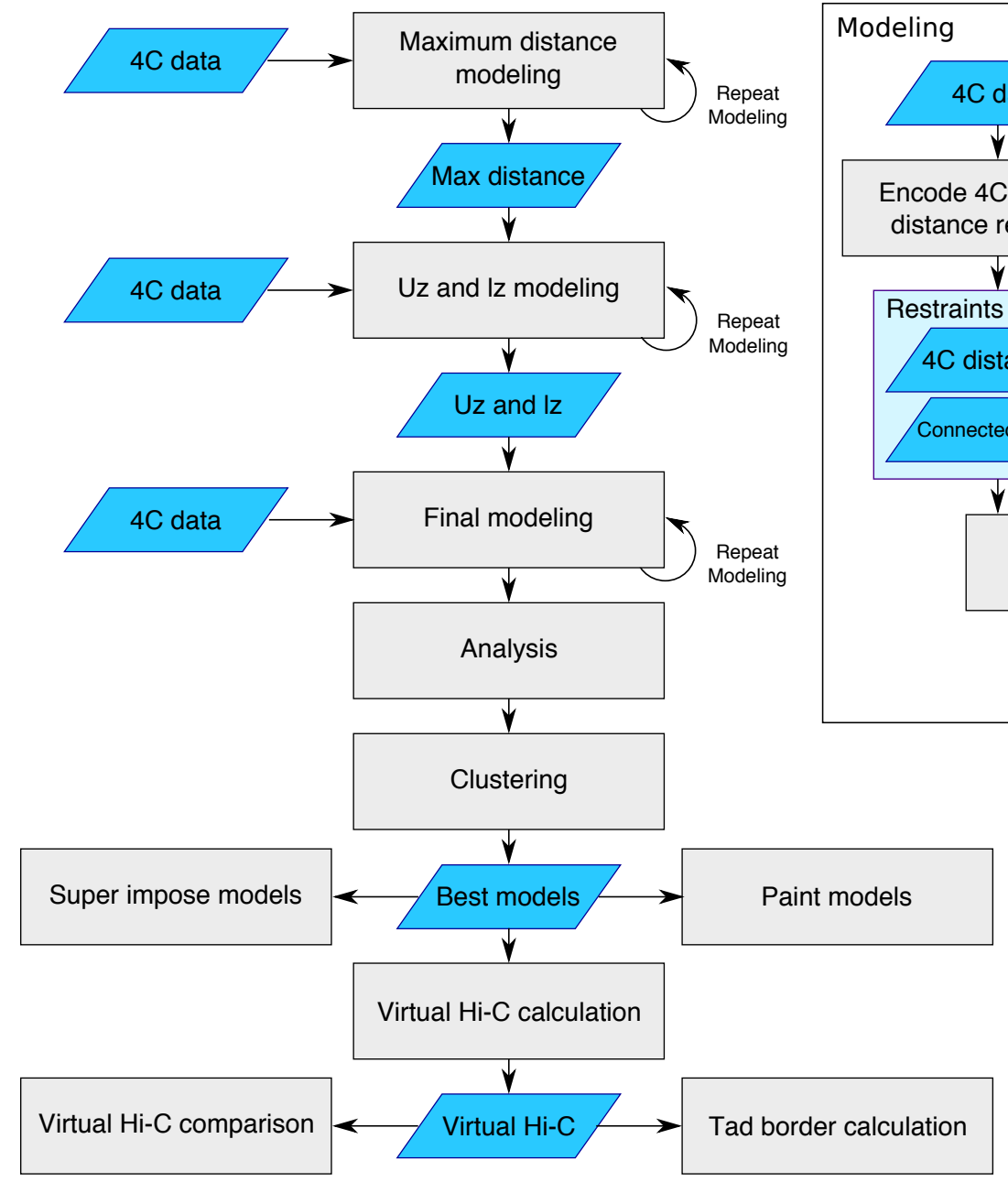

## Modeling

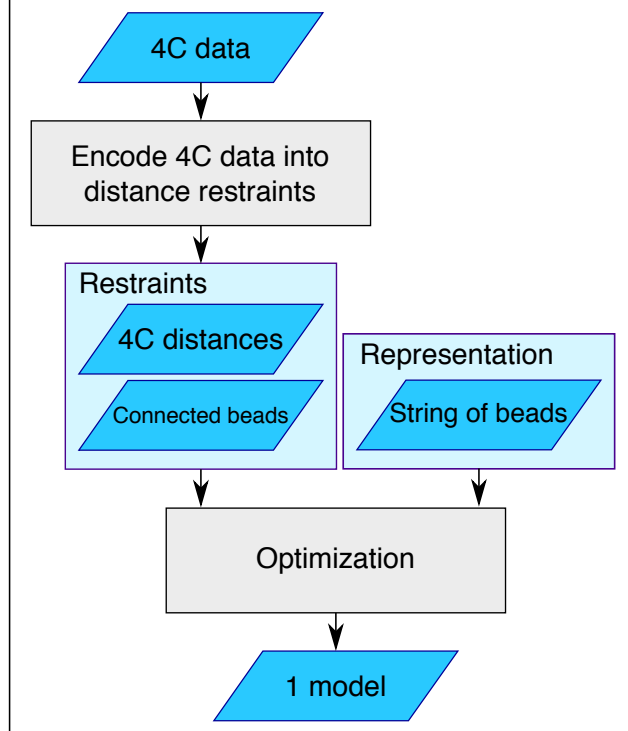

Supplement: S1 Fig — (Pipeline) First, the maximum distance, the upper bound Z-score (uZ) and the lower bound Z-score (lZ) are calculated so these parameters are afterwards used in the final modeling. Then, these models are subjected to an analysis to retrieve the best models and clustered based on their RMSD to distinguish between mirror image models. Best models can also be super imposed, to see structural variability. The representative model can be colored depending on genetic or epigenetic data. Finally, best models are used to generate a virtual Hi-C (vHi-C). Additionally, TAD boundaries can be called in the vHi-Cs and other vHi-Cs can be compared using the scripts provided with the pipeline. (Modeling) The modeling process first encodes the 4C-seq data into restraints. Distance restraints are also used to connect beads. These restraints and the representation of the chromatin fragments as beads are taken into account in the optimization process to generate a single model. (PDF) [file pcbi.1006030.s001.pdf]

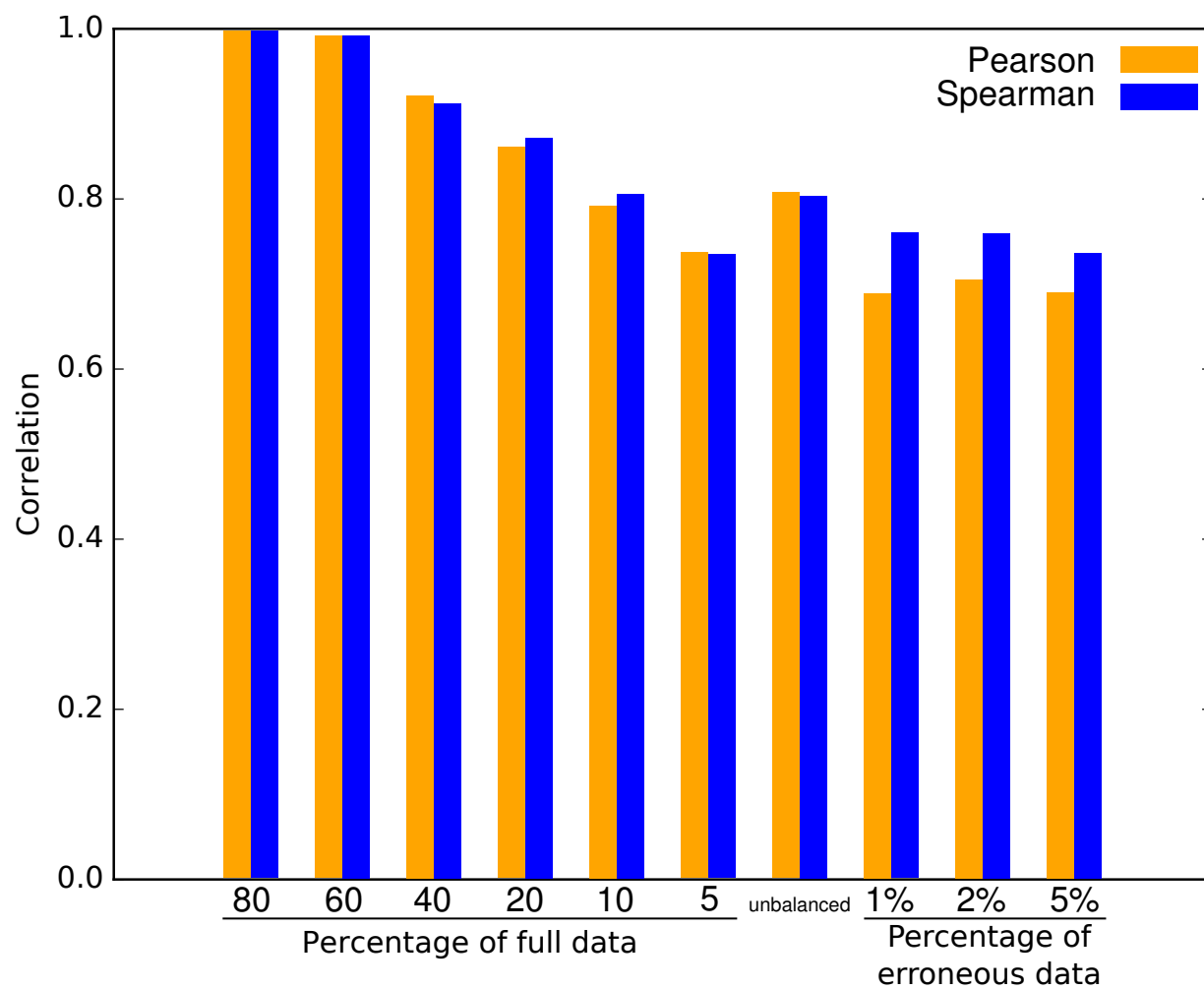

Supplement: S2 Fig — Pearson's and Spearman's correlation between the vHi-C derived from six2a-six3a zebrafish locus models and the vHi-C's of the same locus down-sampling and inserting errors in the 4C data. (PDF) [file pcbi.1006030.s002.pdf]

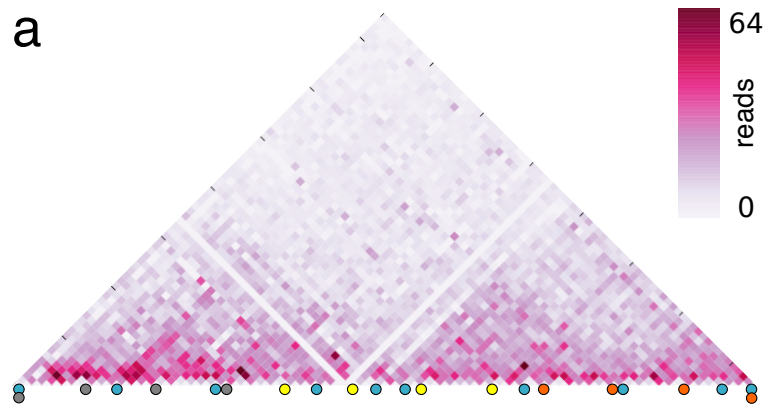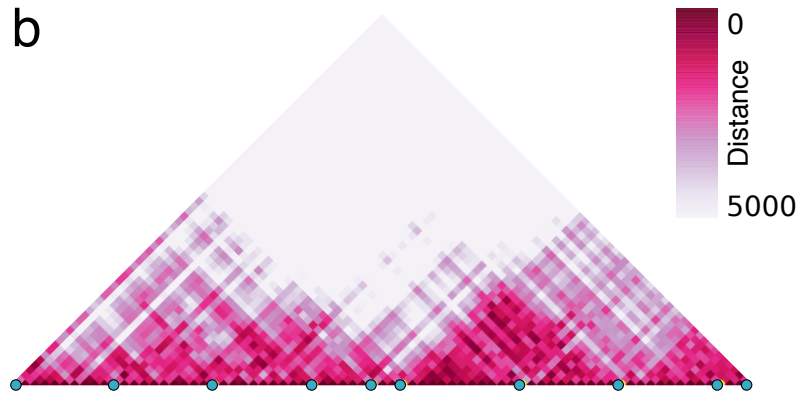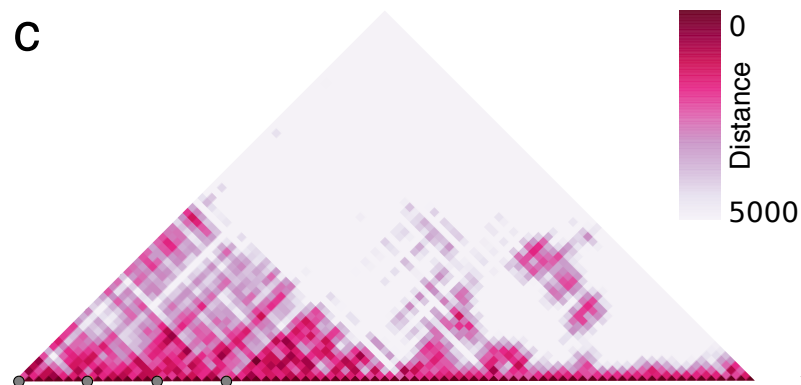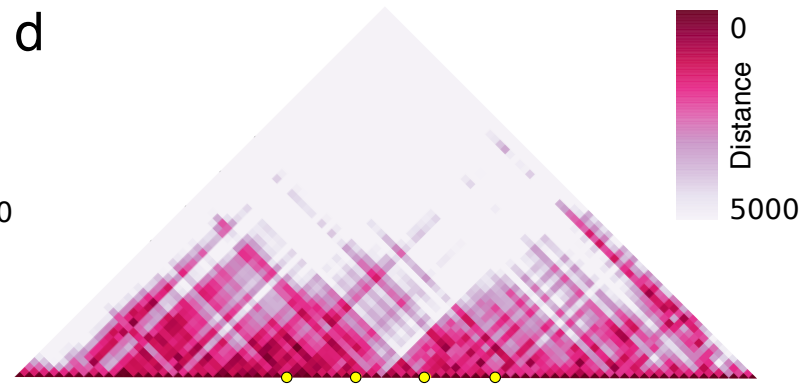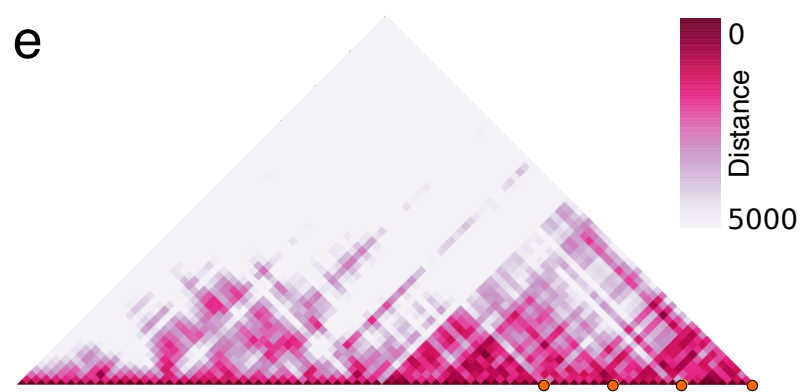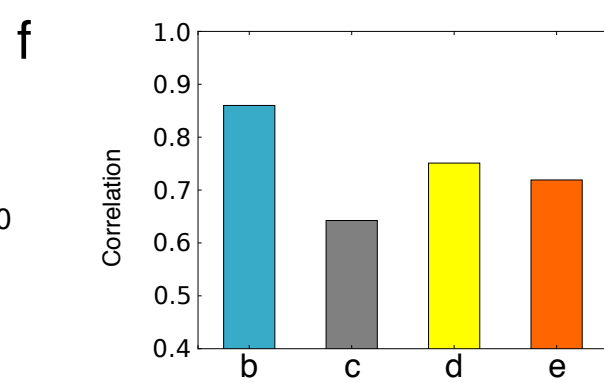

Supplement: S3 Fig — (a) Hi-C of the Six2-Six3 cluster (Gene Expression Omnibus (GEO) accession number GSM862722). (b) vHi-C of the Six2-Six3 cluster. (c,d,e) vHi-Cs of the Six2-Six3 cluster generated with different viewpoints. (f) Spearman's correlation between the Hi-C (a) and the vHi-Cs (b,c,d,e). (PDF) [file pcbi.1006030.s003.pdf]

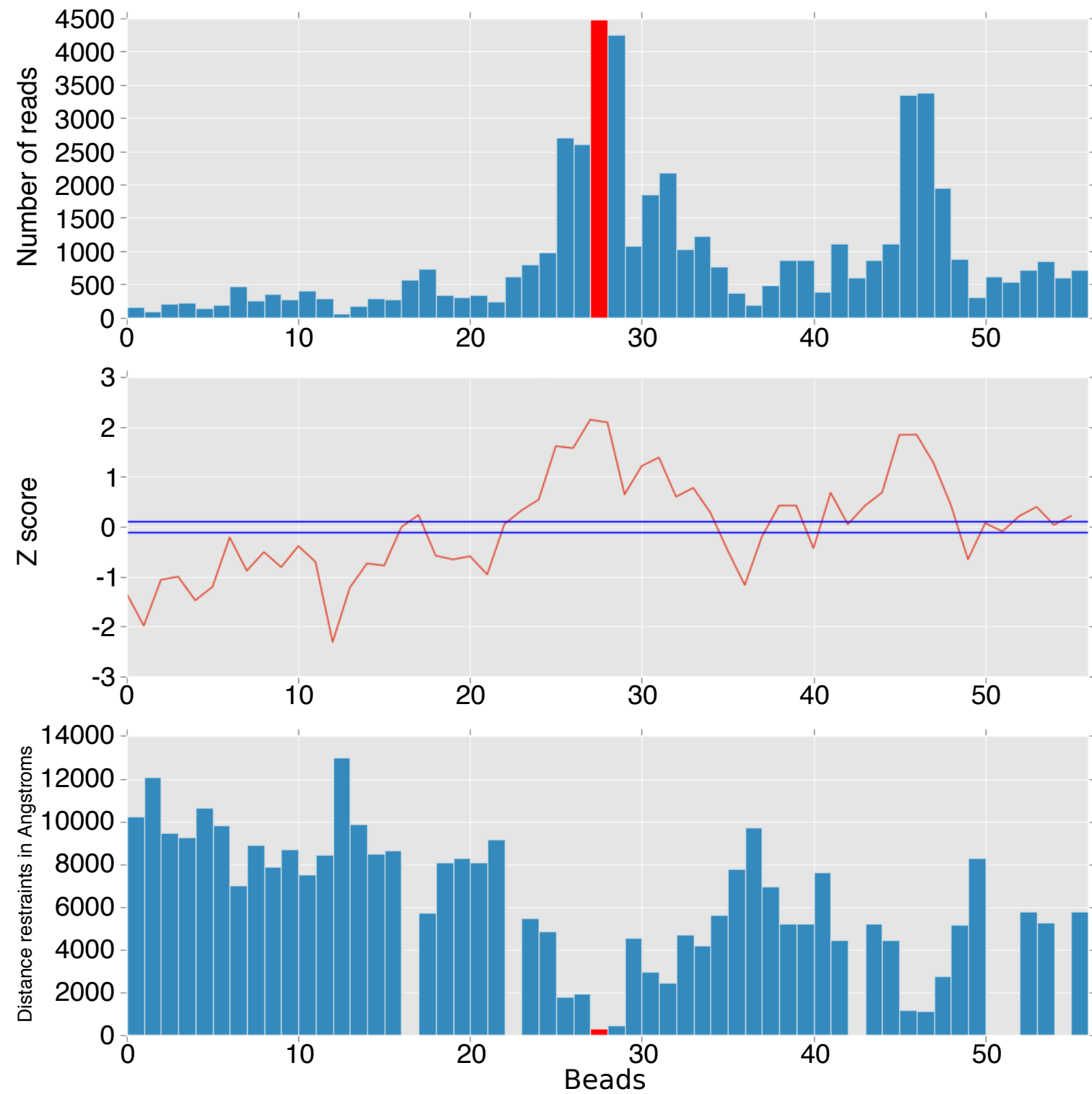

Supplement: S4 Fig — Example corresponds to the Six3 viewpoint in mouse. Top, 4C-seq read counts by bead. Red bar shows the viewpoint. Middle, Z-scores in red corresponding to the read counts from the top panel. Horizontal blue lines indicate the upper bound Z-score and lower bound Z-score. Bottom, Distance restraints encoded from the read counts in the top panel. (PDF) [file pcbi.1006030.s004.pdf]

a

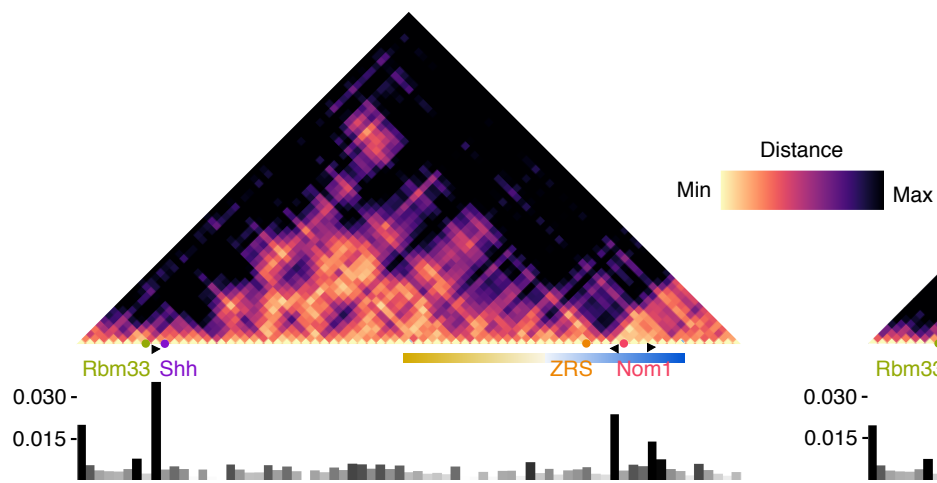

c

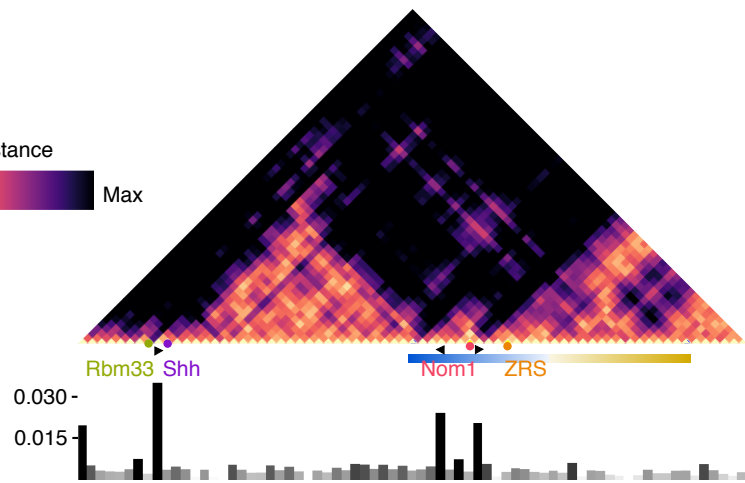

b

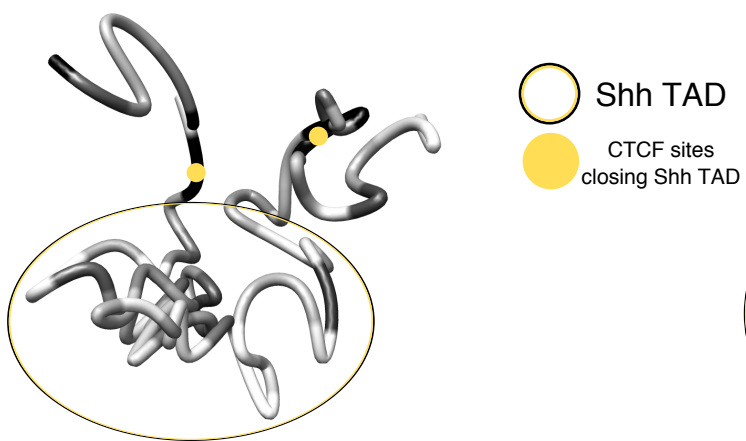

d

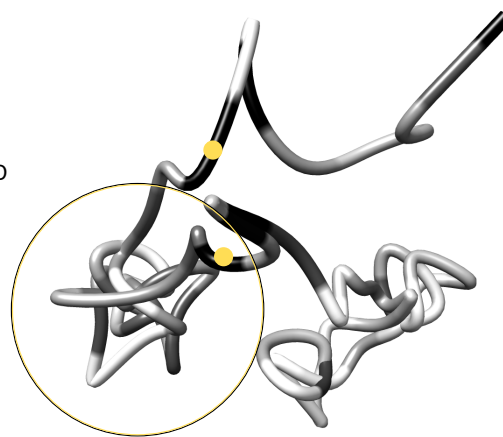

Supplement: S5 Fig — (a) vHi-C of the Shh WT region on top. CTCF Chip-seq data corresponding to the region colored in white-to-black gradient, white for low reads, black for high reads. CTCF sites with highest reads are depicted with oriented triangles. (b) Shh WT representative model colored as in panel (a). Yellow beads represent Shh-TAD boundaries. Shh-TAD is encircled in yellow-black. (c and d) vHi-C, CTCF Chip-seq data and representative model depicted as in (a) and (b). (PDF) [file pcbi.1006030.s005.pdf]

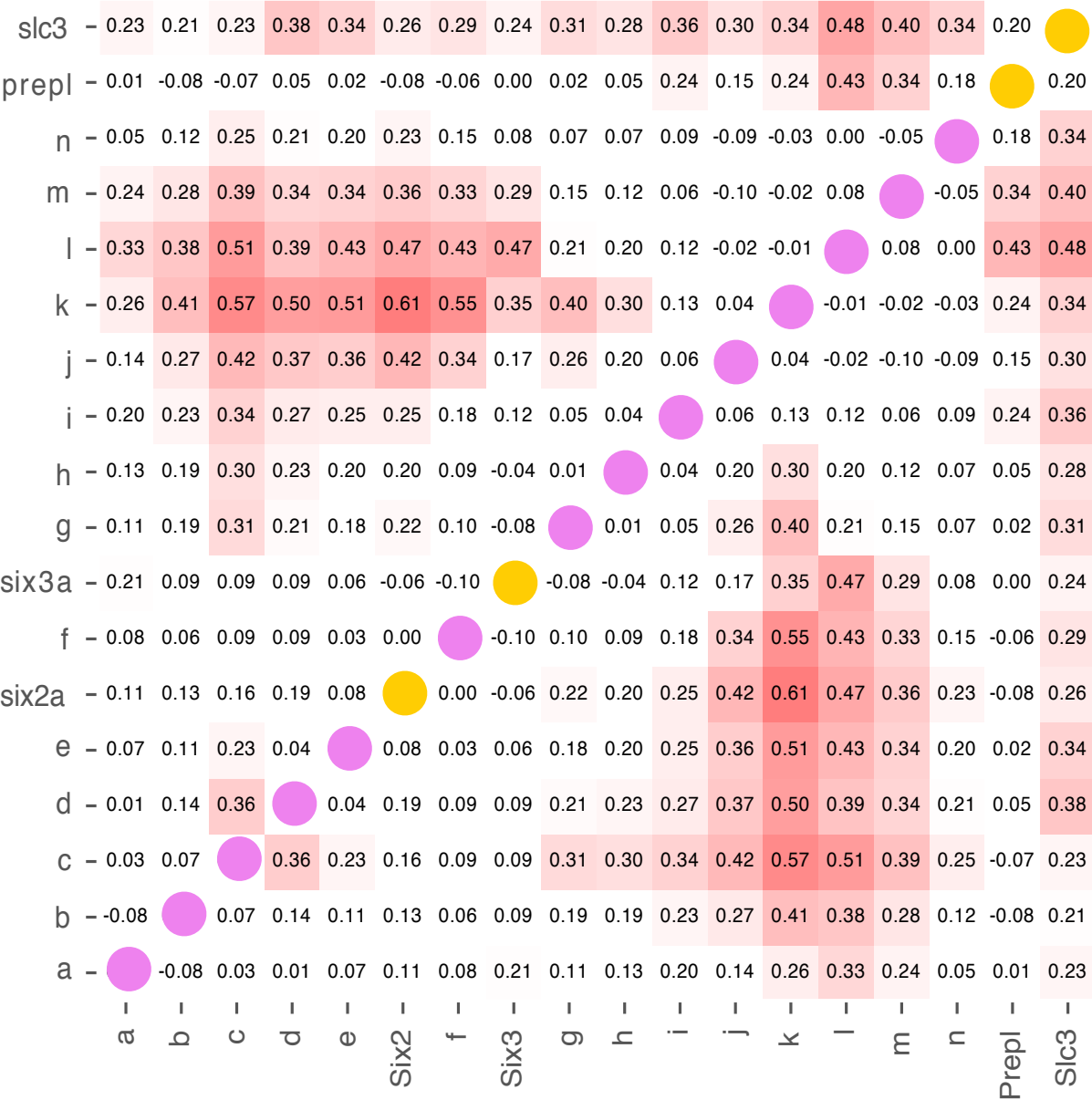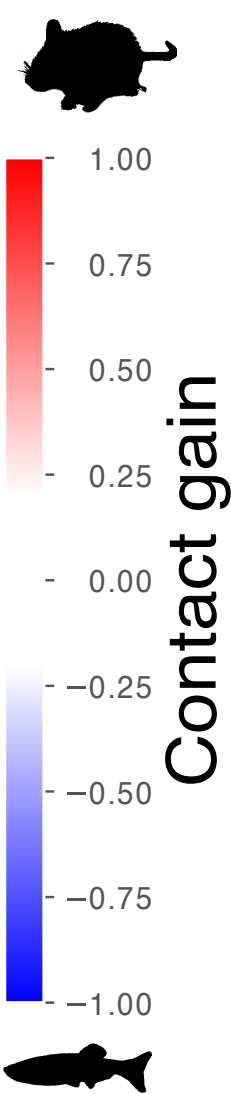

Supplement: S6 Fig — Subtraction heat map of the distance changes between conserved regions in the Six2-Six3 cluster in zebrafish and mouse as explained in Fig 2E. Top triangle corresponds to zebrafish data and bottom triangle to mouse data. Red squares indicate shorter distances in mouse, blue shorter distances in zebrafish. (PDF) [file pcbi.1006030.s006.pdf]

a

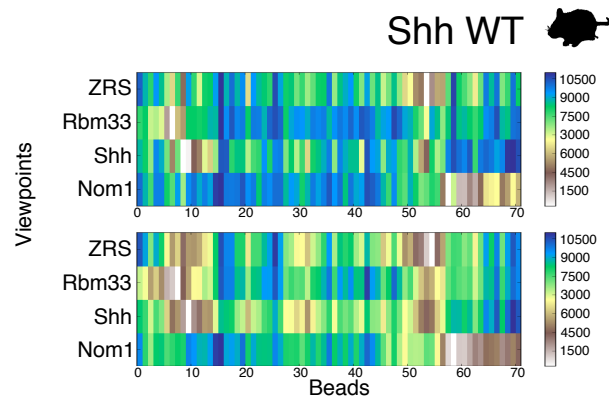

b

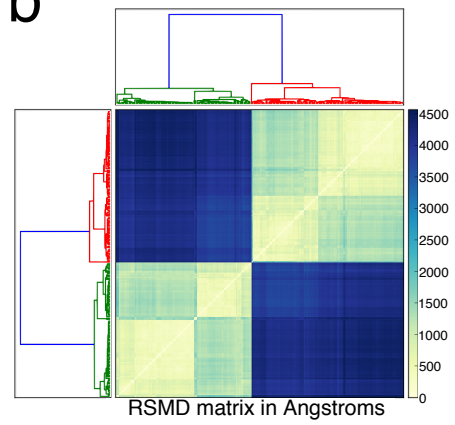

c

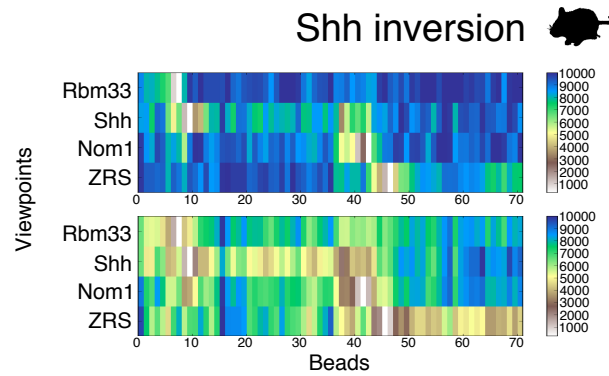

d

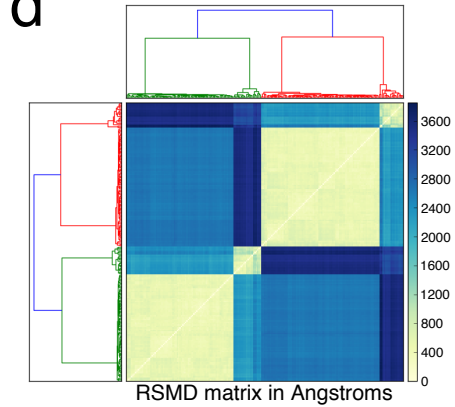

e

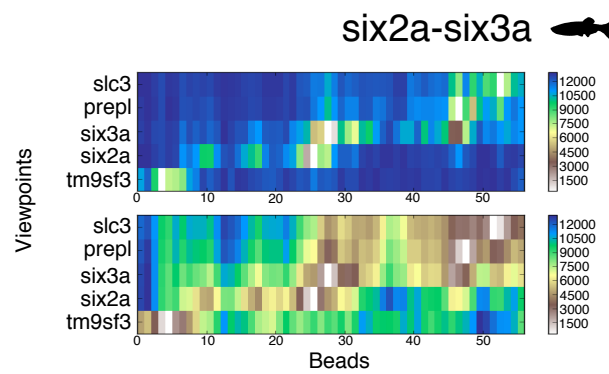

f

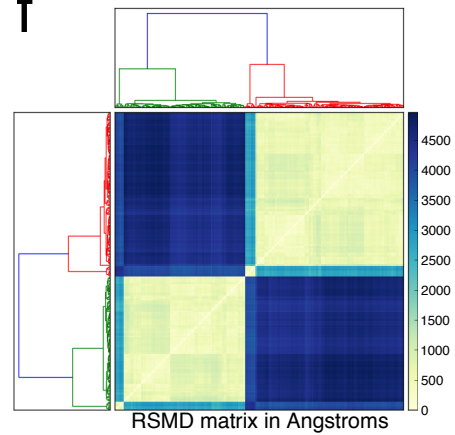

g

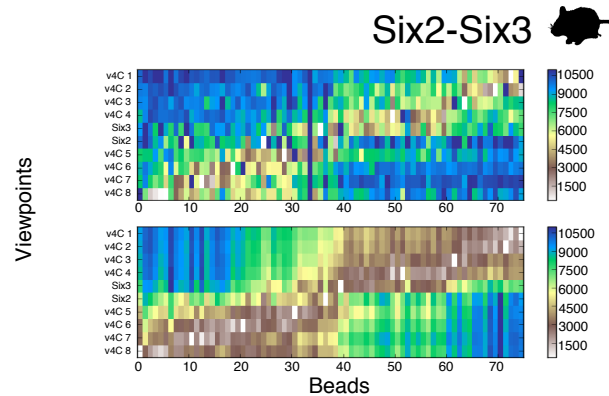

h

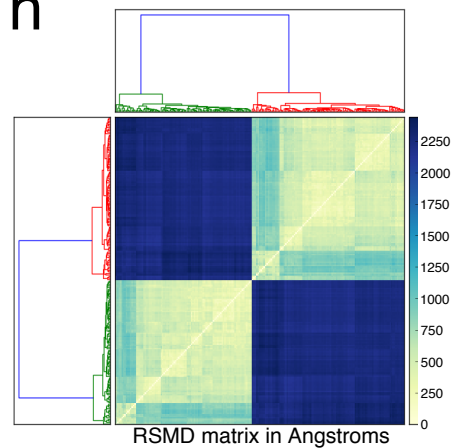

Supplement: S7 Fig — (a, c, e, g) Heat maps comparing the raw 4C-seq data and the mean distances between beads of the models with the best parameters (upper bound Z-score, lower bound Z-score and max distance): Shh WT region: 0.1, -0.1, 11000; Shh inverted region: 0.2, -0.1, 10000; Six2-Six3 cluster in zebrafish: 0.1, -0.1, 13000 and in mouse: 0.2, -0.1, 11000. (b, d, f, h) Heat maps showing 2 clusters in the Shh WT and inverted region and the Six2-Six3 cluster in zebrafish and mouse. The clustering was performed based on the RMSD of the 3D models. (PDF) [file pcbi.1006030.s007.pdf]

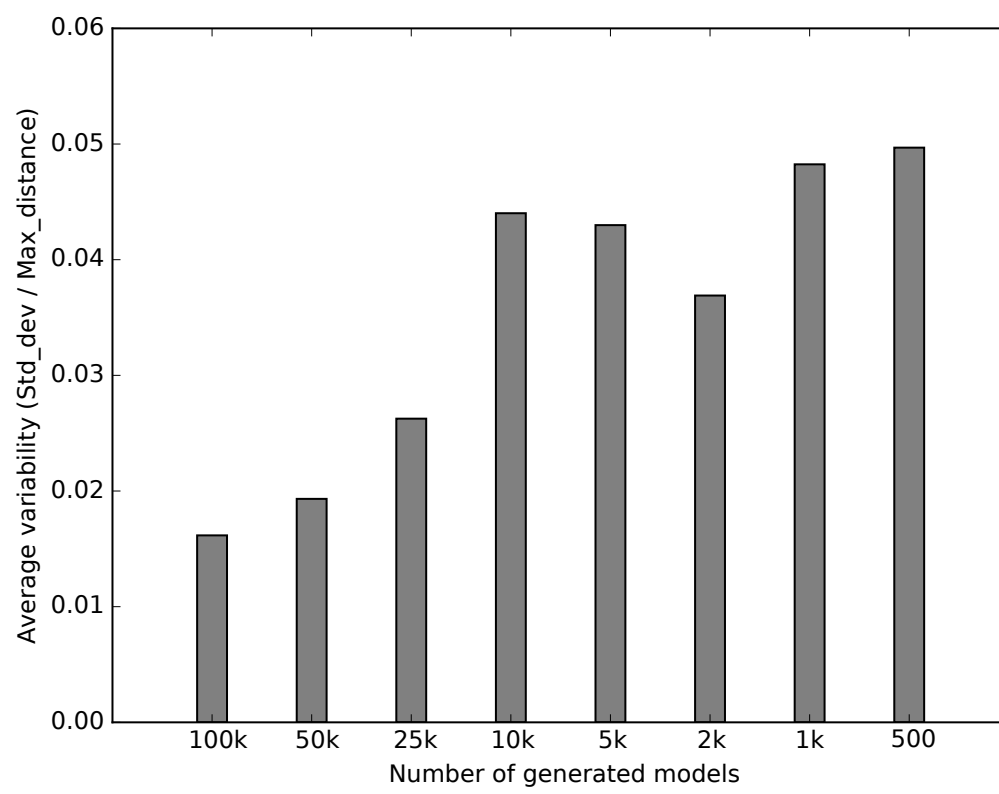

Supplement: S8 Fig — Average variability of the 3D models depending on the sampling. (PDF) [file pcbi.1006030.s008.pdf]

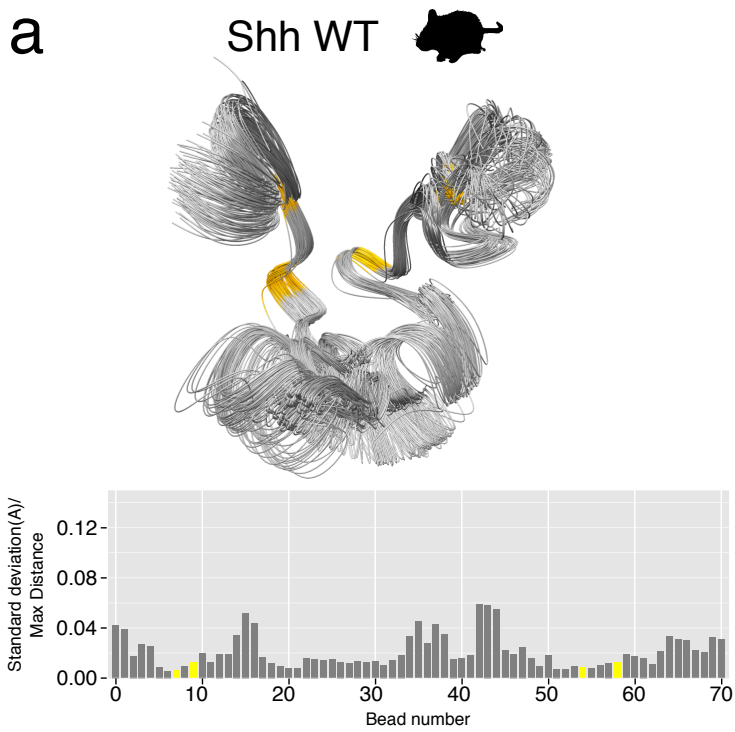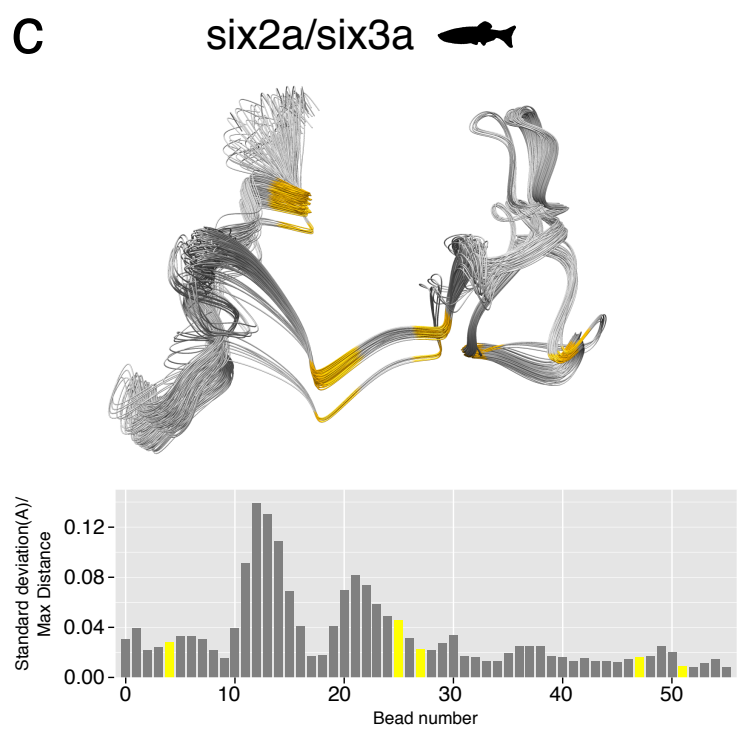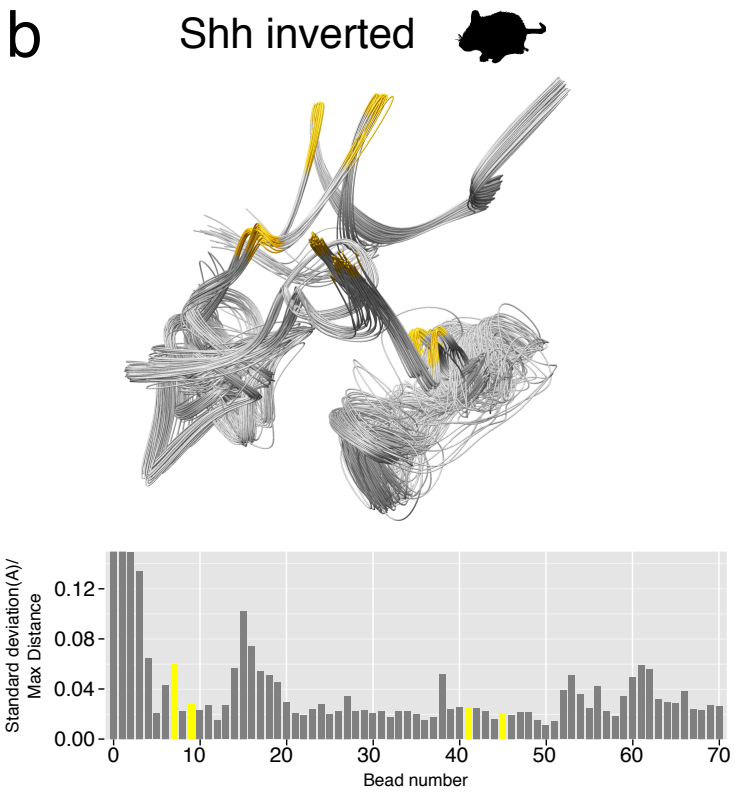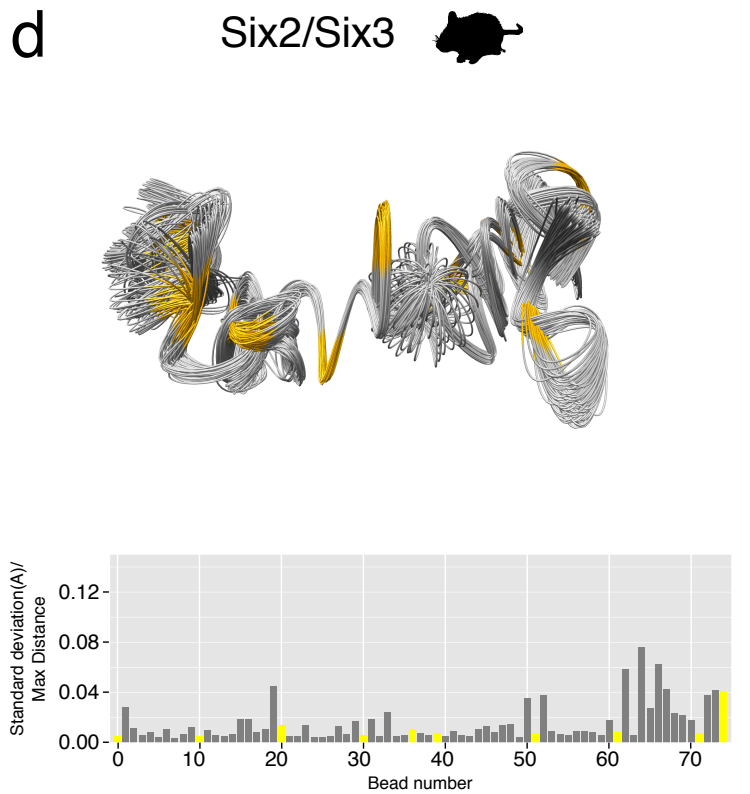

Supplement: S9 Fig — Superposition of 3D models of the biggest cluster after clustering the best models of the Shh WT region and variability of each bead in the cluster showed in standard deviation devided by their maximum distance to show them at scale. (a), Shh inverted region (b), Six2-Six3 cluster in zebrafish (c) and mouse (d). (PDF) [file pcbi.1006030.s009.pdf]
