# Supplementary material for: 4Cin: A computational pipeline for 3D genome modeling and virtual Hi-C analyses from 4C data
Source: PLoS Comput Biol. 2018 Mar 9;14(3):e1006030. doi: 10.1371/journal.pcbi.1006030 (PMC5862518; doi:10.1371/journal.pcbi.1006030)
Supplement: S1 Table — Viewpoints used in the generation of 3D models. (PDF) [file pcbi.1006030.s010.pdf]

Location	Sign	Motif	Clover score	Bead number (WT)	Sign (WT)	Bead number (INV(6-C2))	Sign (INV(6-C2))
chr5:28796760-28796774	+	gcaccagggggaggt	7.19	8	+	8	+
chr5:29705888-29705902	-	gatgccctcttctgg	7.32	57	-	42	+
chr5:29707165-29707179	-	ggtgccagctcctgg	7.19	57	-	42	+
chr5:29711103-29711117	-	ggtaccctcttctgg	7.06	57	-	42	+
chr5:29717148-29717162	+	ccagaagagggcatc	7.24	57	+	42	-
chr5:29794041-29794055	+	ccagaagagggcatc	7.24	61	+	38	-
chr5:29798024-29798038	+	ccaaaagagggcgcc	7.73	61	+	38	-
chr5:29806209-29806223	-	gatgccctctcctgg	7.28	61	-	38	+
