# Supplementary material for: 4Cin: A computational pipeline for 3D genome modeling and virtual Hi-C analyses from 4C data
Source: PLoS Comput Biol. 2018 Mar 9;14(3):e1006030. doi: 10.1371/journal.pcbi.1006030 (PMC5862518; doi:10.1371/journal.pcbi.1006030)
Supplement: S2 Table — Conserved regions and genes between Zebrafish and Mouse in the Six2-Six3 region. (PDF) [file pcbi.1006030.s011.pdf]

Type	Position	Bead number
Enhancer	chr5:28785355-28786651	42
	chr5:28802630-28803728	45
	chr5:28889675-28890477	68
	chr5:29111755-29112865	135
	chr5:29202010-29203160	161
	chr5:29219233-29219616	166
	chr5:29412199-29413107	217
	chr5:29472668-29473966	232
	chr5:29514035-29515033	241
	Chr5:29538538-29539344	247
	chr5:29576796-29578633	257
	chr5:29641382-29643298	272
No Expression Sensor	chr5:29077184	124
	chr5:29094638	130
	chr5:29297175	188
	chr5:29430394	221
Low Expression Sensor	chr5:28993084	97
	chr5:29089727	128
	chr5:29171252	153
	chr5:29208077	162
	chr5:29209269	163
	chr5:29390735	212
	chr5:29466562	230
High Expression Sensor	chr5:28948195	84
	chr5:29159936	149
	chr5:29260046	179
	chr5:29403806 and chr5:29405415	215
	chr5:29413901 and chr5:29415912	218
	chr5:29420191	219
	chr5:29445731	226
	chr5:29451881 and chr5:29452662	227
	chr5:29463020	229
	chr5:29467666 and chr5:29470352	231
	chr5:29473391	232
	chr5:29533044	246
	chr5:29550146	250
