# Supplementary material for: 4Cin: A computational pipeline for 3D genome modeling and virtual Hi-C analyses from 4C data
Source: PLoS Comput Biol. 2018 Mar 9;14(3):e1006030. doi: 10.1371/journal.pcbi.1006030 (PMC5862518; doi:10.1371/journal.pcbi.1006030)
Supplement: S3 Table — Location and sign of CTCF binding sites. (PDF) [file pcbi.1006030.s012.pdf]

Viewpoint Name (Zebrafish/Mouse)	Position Zebrafish	Bead number representing viewpoint Zebrafish	Position Mouse	Bead number representing viewpoint Mouse
a	chr13:9269884-9270183	6	chr17:86529466-86529615	62
b	chr13:9295309-9295524	8	chr17:86497776-86497977	60
c	chr13:9346911-9347326	1	chr17:86429477-86429864	57
d	chr13:9705043-9705579	20	chr17:86222517-86223016	47
e	chr13:9763311-9764531	23	chr17:86147425-86148444	43
six2a/Six2	chr13:9797970	25	chr17:86080000	39
f	chr13:9821640-9821721	26	chr17:86028337-86028418	37
six3a/Six3	chr13:9826837	27	chr17:86020000	36
g	chr13:9892892-9892942	30	chr17:85920377-85920427	32
h	chr13:9904784-9904990	31	chr17:85885652-85885878	30
i	chr13:9940213-9940824	32	chr17:85853966-85854458	28
j	chr13:9958684-9958818	34	chr17:85832300-85832434	27
k	chr13:9979405-9979935	35	chr17:85793540-85794012	25
l	chr13:10025682-10026111	38	chr17:85776197-85776601	24
m	chr13:10064808-10065017	40	chr17:85719504-85719710	21
n	chr13:10073086-10073446	41	chr17:85694199-85694560	20
Prepl	chr13:10186453	47	chr17:85462817	9
Slc3a	chr13:10215863	51	chr17:85427687	7
