# Supplementary material for: 4Cin: A computational pipeline for 3D genome modeling and virtual Hi-C analyses from 4C data
Source: PLoS Comput Biol. 2018 Mar 9;14(3):e1006030. doi: 10.1371/journal.pcbi.1006030 (PMC5862518; doi:10.1371/journal.pcbi.1006030)
Supplement: S4 Table — Sensor probes and enhancers in the Shh region. (PDF) [file pcbi.1006030.s013.pdf]

Locus	Viewpoint Name	Position	Bead number representing viewpoint
	tm9sf3	chr13:9240919	4
six2a/six3a	six2a	chr13:9797970	25
zebrafish	six3a	chr13:9826837	27
	prepl	chr13:10186394	47
	slac3a	chr13:10237450	51
	85300000	chr17:85300000	0
	85500000	chr17:85500000	10
	85700000	chr17:85700000	20
	85900000	chr17:85900000	30
Six3/Six2	86020000 (Six3)	chr17:86020000	36
mouse	86080000 (Six2)	chr17:86080000	39
	86320000	chr17:86320000	51
	86520000	chr17:86520000	61
	86720000	chr17:86720000	71
	86780000	chr17:86780000	74
	Rbm33	chr5:28749055	7
Shh WT	Shh	chr5:28804534	9
mouse	ZRS	chr5:29637990	54
	Nom1	chr5:29731026	58
	Rbm33	chr5:28749055	7
Shh INV	Shh	chr5:28804534	9
mouse	ZRS	chr5:29476620	45
	Nom1	chr5:29383581	40
